# Lipid nanoparticles as active biointerfaces: From membrane interaction to systemic dysregulation

**DOI:** 10.1016/j.apsb.2026.07.001

**Published:** 2026-07-06

**Authors:** Falko Seger, L. Maria Gutschi, Stephanie Seneff

**Affiliations:** a Independent Researcher, Berlin 10117, Germany; b Pharmacy Consultant, Retired, Ottawa K1P 1C1, Canada; c Computer Science and Artificial Intelligence Laboratory, MIT, Cambridge, MA 02139, USA

**Keywords:** Lipid nanoparticles, Ionizable lipids, mRNA therapeutics, Oxidative stress, Phosphatidylinositol cycle, Membrane disruption, Signaling cascades, Cell homeostasis

## Abstract

Lipid nanoparticles (LNPs) are central to modern mRNA therapeutics, including COVID-19 vaccines. Far from passive carriers, their ionizable lipids actively interact with cellular membranes. Evidence from cellular, transcriptomic, and proteomic studies indicates that LNPs, with or without nucleic acid, alter gene and protein expression, thereby initiating inflammatory, detoxification, and stress responses at the membrane. Key pathways affected include lipid metabolism and detoxification, with roles for Peroxisome Proliferator-Activated Receptor Gamma (PPAR*γ*) and cytochrome P450 enzymes. We hypothesize that the phosphatidylinositol (PI) cycle is the primary site of LNP-induced perturbations, regulating membrane restructuring and organelle trafficking during endocytosis. Disruption of this cycle triggers downstream signaling cascades, including nuclear factor kappa B (NF-*κ*B), mitogen-activated protein kinases (MAPKs), Janus kinase/signal transducers and activators of transcription (JAK/STAT), and mechanistic target of rapamycin (mTOR). We term this systemic effect lipid-nanoparticle-driven membrane dysfunction (L-DMD), characterized by dysregulated cellular communication, stress responses, and energy balance. This review provides a mechanistic framework for understanding the persistent biological effects of modified modRNA-LNP exposure and emphasizes a systems-level intracellular perspective.

## Graphical abstract

Lipid nanoparticles, with or without mRNA, disrupt membranes by interfering with the phosphatidylinositol cycle, activating downstream signaling pathways and potentially driving systemic signaling dysregulation.

**Figure:**
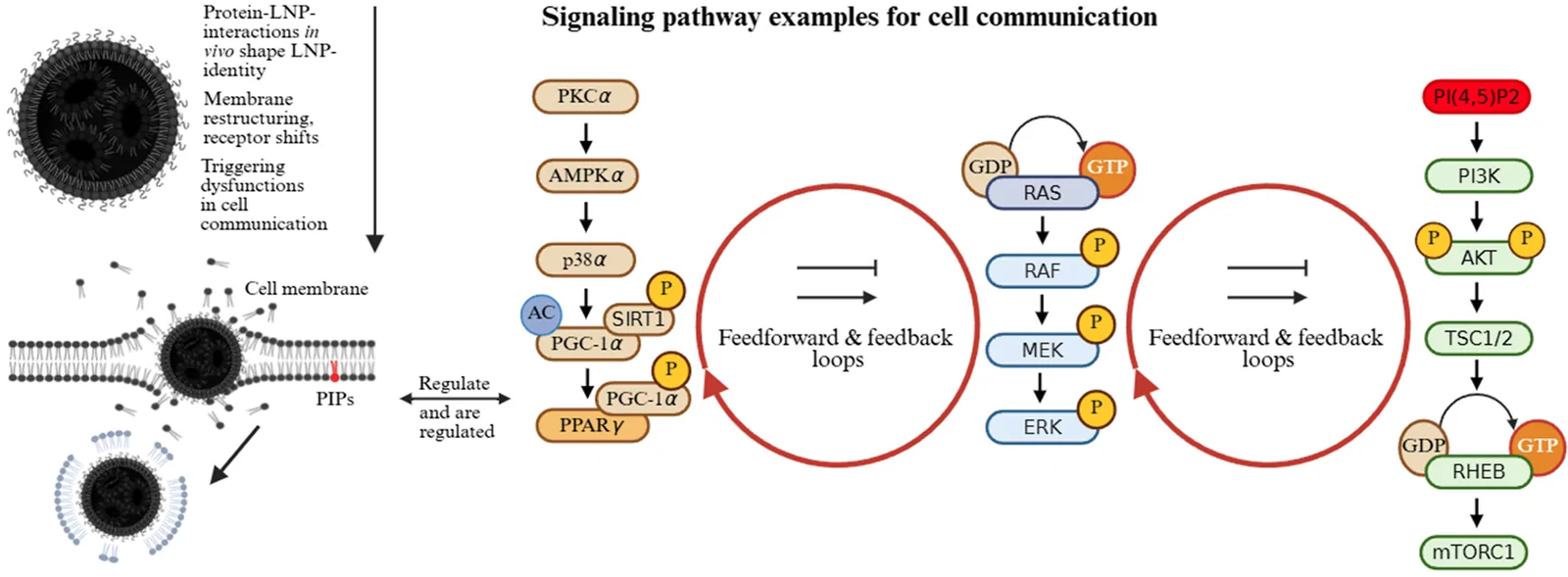

## 1 Introduction

Lipid nanoparticles (LNPs) used in mRNA vaccine technology are engineered to resemble low-density lipoprotein (LDL) particles, thereby improving cellular penetration and facilitating endosomal escape^1^. Four distinct lipid types comprise LNPs: cationic ionizable lipids, cholesterol, phospholipids, and PEGylated lipids. Cationic ionizable lipids aid endosomal escape, cholesterol stabilizes the membrane and supports fusion, and phospholipids stabilize the LNP^2^. PEGylation, the addition of polyethylene glycol (PEG), masks the lipid particle's cationic surface charge and provides a hydrophilic stealth coating. Ionizable lipids interact with negatively charged endosomal phospholipids by forming cone-shaped ion pairs^3^. PEG coatings shield the surface from aggregation, opsonization, and phagocytosis^4^.

**Figure 1:**
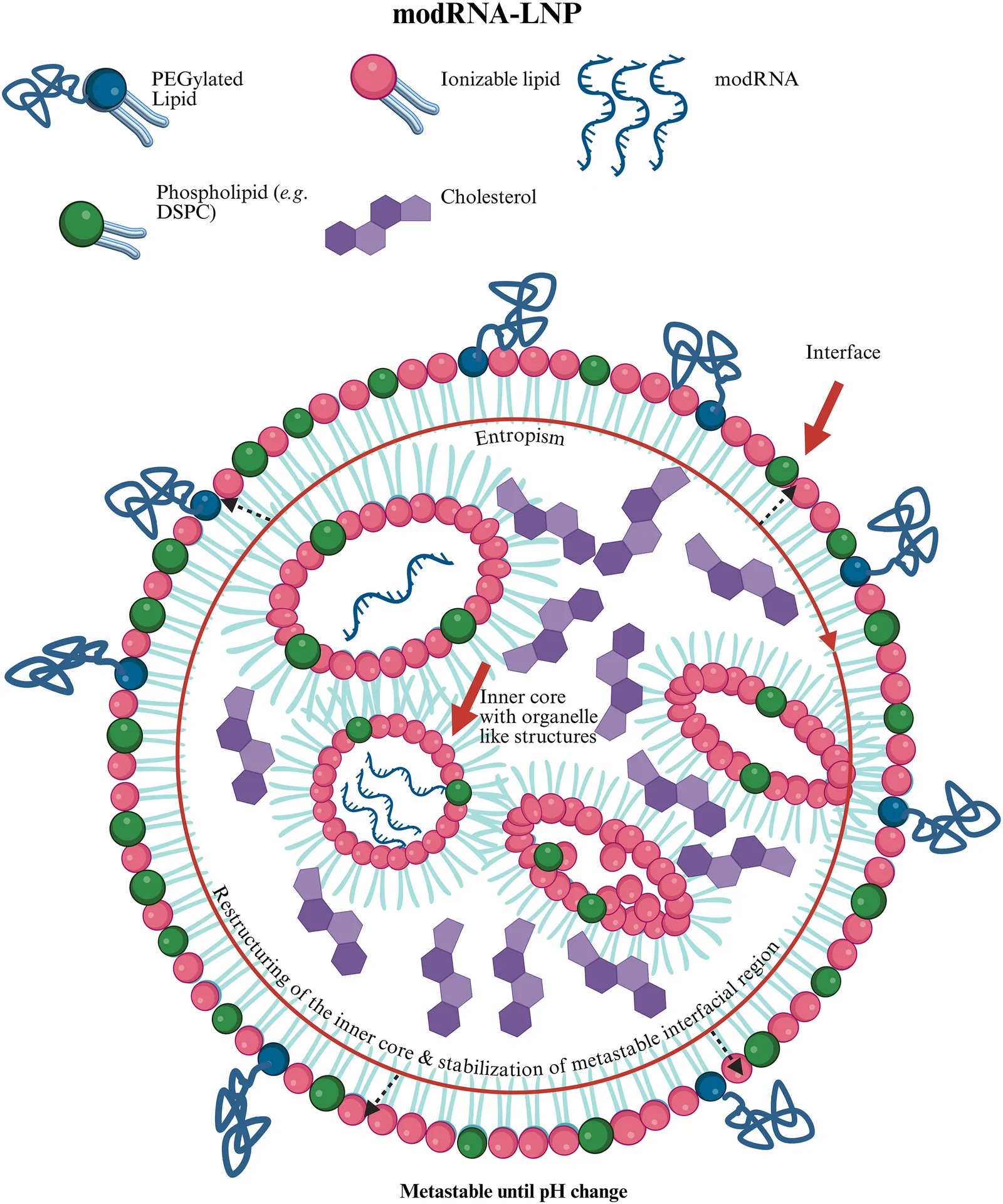
Schematic structural representation of an LNP (*via* Cryo EM). Diverse weak-loaded forces keep the LNP metastable. Created in BioRender. Seger, F. (2026). https://BioRender.com/ysmgilk.

While LNPs are recognized as biointeractive entities that integrate into cellular membranes and influence membrane structure, leaflet asymmetry, and local electrostatics, current pharmacodynamic and toxicological frameworks treat lipid-mediated effects as secondary or transient to the resulting protein expression (Fig. 1). These frameworks focus primarily on nucleic acid payload, generalized innate immune responses, and protein expression, without linking membrane-level perturbations to downstream signaling dysregulation. We propose lipid-nanoparticle-driven membrane dysfunction (L-DMD), a hypothesis centered on phosphatidylinositol (PI) cycle perturbations, as a unifying mechanistic framework connecting structural membrane changes to possible persistent alterations in intracellular signaling pathways, and consequently at the transcriptional level, including nuclear factor kappa B (NF-*κ*B), mitogen-activated protein kinases (MAPKs), Janus kinase/signal transducers and activators of transcription (JAK/STAT), and mechanistic target of rapamycin complex 1/2 (mTORC1/2).

### 1.1 L-DMD–a rational hypothesis

LNPs are often seen as delivery vehicles for nucleic acids, but their biological activity goes beyond payloaded transport. Their physicochemical properties, particularly those of ionizable lipids and PEGylated lipids, render them inherently biointeractive (Fig. 1). The ionizable lipids are engineered to undergo charge transitions in response to their local environment^5^, enabling membrane interaction, bilayer penetration, and endosomal escape^6^^,^^7^. We propose LNPs act as active modifiers of membrane structure and function rather than passive carriers; cryogenic transmission electron microscopy (Cryo-TEM) indicates that LNPs lack a hollow aqueous core or stable internal membrane bilayers^2^^,^^8^. Instead, the encapsulated ribonucleic acid is intimately associated with ionizable lipids through electrostatic interactions^9^. The negatively charged phosphate backbone of the ribonucleic acid interacts directly with positively charged or protonatable lipid headgroups, forming a compact, disordered lipid-nucleic acid core^10^ that reflects a metastable, non-crystalline lipid-nucleic acid assembly capable of structural reorganization, including bleb formation^11^.

LNPs are stabilized predominantly by weak, non-covalent interactions, including electrostatic attraction, van der Waals forces, and hydrophobic effects^8^^,^^10^. No single interaction maintains structural integrity; stability arises from the collective interactions and flexibility of lipids and nucleic acids. The core is best seen as a dynamic network stabilized by the high configurational freedom of its components rather than from fixed architectural elements^12^.

Consequently, the outer lipid shell forms through spontaneous self-organization of lipids at the interface, not *via* an internal scaffold or layered membrane system^2^. This structure is sufficiently stable to permit formulation, storage, and systemic transport^13^, yet it remains deliberately metastable^14^. Its metastability enables structural rearrangements in response to environmental cues, such as pH shifts or membrane contact, thereby facilitating cellular uptake and release of nucleic acid payload.

Given these properties, LNPs are best described as supramolecular assemblies rather than fixed molecules^15^. Their behaviour reflects their multiparticulate, dynamic, and colloidal nature, with structure arising from collective interactions^16^. This distinction is not merely semantic; it has implications for how LNPs are perceived, their interactions with biological systems, their responses to environmental stimuli, and their structural transitions *in vivo.*

Having outlined key structural and organizational features of LNPs, it is necessary to briefly discuss how these structures function *in vivo*. Such a discussion is essential to bridge the gap between formulation design concepts derived from physicochemical considerations and the actual biological behavior of LNPs after administration.

In biological environments, LNPs are exposed to complex and heterogeneous conditions, including variable pH, ionic strength, protein coronas, and membrane interfaces. Under these conditions, their colloidal organization enables adaptive responses that may not be readily predictable from static structural models alone. As a result, LNPs may exhibit emergent nonlinear biological effects stemming from the interplay among particle composition, membrane interactions, and cellular context. Understanding these behaviors requires explicit consideration of both their physicochemical design principles and their dynamic reorganization *in vivo*. This structural characterization describes LNPs in isolation. To fully evaluate their biological impact, it should be considered how these metastable assemblies may behave in the complex environment of living systems.

### 1.2 The special properties of ionizable lipids

Central to this *in vivo* reorganization may be the ionizable lipids themselves, whose unique physicochemical properties could drive membrane interaction and integration. At membrane interfaces, the metastable behaviour of LNPs decomposes into the actions of individual lipids, which mediate interactions with cell membranes. Recent studies show that ionizable lipids can directly integrate into phospholipid bilayers^17^^,^^18^. Molecular dynamics simulations reveal that ionizable lipid nanodroplets merge spontaneously with model membranes, with insertion of lipid components into the bilayer rather than transient surface adsorption^17^. These simplified models isolate key physicochemical interactions that are otherwise masked in fully biological systems. Free energy profiles indicate several clinically used ionizable lipids favor partitioning into phospholipid membranes, with insertion depth and stability depending on lipid chemistry, bilayer composition, and phase state^18^.

These findings are in keeping with the structure-function relationships described by Atmuri et al.^19^, who demonstrated experimentally that molecular geometry (*i.e.*, branched *vs*. linear tails, ionizable head-group, p*K*a^1^, and linker biodegradability) can influence bilayer disruption and retention. Notably, membranes with higher phase-transition temperatures and greater lipid order (such as liquid-ordered cholesterol-enriched domains) are more susceptible to penetration by ionizable lipids, indicating that the membrane's physical state critically determines lipid integration. Importantly, such insertion represents the expected behavior of a metastable supramolecular assembly undergoing dynamic interactions with the lipid bilayer.

Ionizable lipids may cause lasting changes in membrane organization beyond insertion, affecting leaflet asymmetry, cholesterol distribution, lipid packing density, and membrane fluidity, often locally, at signaling microdomains^17^. These perturbations may alter the electrostatic and dielectric properties of the inner leaflet^20^, crucial for membrane signaling. Since phosphoinositide (PIP)-dependent signaling relies on membrane-protein interactions and exhibits^21^ modest but long-lived perturbations in lipid packing, local charge density can be sufficient to destabilize them^22^.

Building on this conceptual gap, we present our L-DMD hypothesis, introducing how lipid-induced membrane perturbations may drive system-wide signaling dysregulation.

### 1.3 Powerful signaling effects of phosphoinositides (PIPs)

Within this context, phosphoinositides (PIPs; also referred to as PtdInsP) play a central mechanistic role. Phosphatidylinositol-4,5-bisphosphate (PI(4,5)P_2_) and Phosphatidylinositol-3,4,5-trisphosphate (PI(3,4,5)P_3_) are low-abundance lipids that nonetheless control a disproportionate share of cellular membrane proximal–proximal signaling processes^21^, ^22^, ^23^, ^24^. They serve as spatial organizers for second messengers, regulate ion channels and vesicular trafficking, organize actin-membrane interactions, and act as obligatory cofactors for enzymes such as phospholipase C and phosphoinositide 3-kinase (PI3K)^21^. Importantly, their signaling function is governed not by bulk concentration but by electrostatics, dielectric interactions, spatial availability, and orientation within the inner plasma membrane.

PI(4,5)P_2_ organization is dictated by electrostatic interactions between its highly negative headgroup and clusters of basic residues in intrinsically disordered protein domains. Proteins such as myristoylated alanine-rich C kinase substrate (MARCKS), epithelial sodium channel (ENaC) and growth-associated protein 43 (GAP-43) reversibly bind multiple PI(4,5P)_2_ molecules through multivalent electrostatic binding, forming separate lipid pools^21^^,^^25^^,^^26^. This dynamic system depends on input: increased calcium promotes calcium-calmodulin binding, displaces proteins, and releases sequestered lipids, while phosphorylation neutralizes positive charges and releases PI(4,5)P_2_. Consequently, membrane surface charge and lipid packing regulate the formation of signaling complexes productively^27^.

Perturbations of membrane electrostatics therefore have immediate and predictable consequences for PIP-dependent signaling. Integration of ionizable lipids into the plasma membrane is expected to alter local charge density, modify lipid headgroup spacing, and change the effective dielectric environment experienced by PIPs and their binding partners. Even modest shifts in these parameters can disrupt the balance between sequestration and release of PI(4,5)P_2_ and PI(3,4,5)P_3_, thereby reshaping the spatial logic of membrane signaling^28^. Given that ionizable lipids demonstrably integrate into membranes and alter local charge density (as discussed in Section 1.2), such perturbations represent a mechanistically plausible and testable scenario warranting investigation.

From this electrostatic perspective, signal-regulatory disorder represents a direct physicochemical consequence rather than a secondary biological effect. Signaling pathways depend on precise PIPs positioning, including PI3K and serine/threonine-specific protein kinases (PKB, also known and referred to as AKT). Phospholipase C-dependent calcium signaling, small GTPase activation, and actin-regulating cascades are inherently susceptible to disruptions in membrane lipid organization because these pathways are organized at the membrane through lipid–protein co-assembly rather than isolated protein–protein interactions. Therefore, alterations in lipid availability propagate across multiple signaling axes simultaneously^29^.

Despite advances in understanding LNP physicochemical behaviour and membrane interactions, current pharmacodynamic and toxicological frameworks largely treat LNPs as inert delivery vehicles, attributing adverse effects primarily to the encoded antigen (*e.g.*, the spike protein) or non-specific innate immune activation (*e.g.*, Toll-like receptor (TLR) activation^30^). This view may underestimate the direct bioactive potential of lipid components, both individually and in assemblies, particularly their ability to induce sustained alterations in membrane electrostatics and PIP organization: the integration of exogenous ionizable lipids into cell membranes may lead to durable changes in local charge density and lipid packing, disrupting spatially limited PI/PI(4,5)P_2_/PI(3,4,5)P_3_ pools exceeding thresholds, subsequently dysregulating multiple membrane–proximal signaling pathways (*e.g.*, PI3K/Akt/mTORC1) axis^31^, phospholipase C-calcium signaling^32^^,^^33^, small guanosine triphosphatases (GTPases)^34^, and actin regulatory cascades^35^ in a cell-type- and context-dependent manner.

L-DMD describes physicochemical disruption of membrane-organized signaling rather than nonspecific toxicity or antigen-driven immune activation. Our hypothesis predicts comparable pathway effects from empty and payloaded LNPs, dependence on ionizable lipid partitioning, mitigation *via* restoration of PI(4,5)P_2_/PI(3,4,5)P_3_ homeostasis, and cumulative effects after repeated exposure. The following sections review supporting evidence for membrane integration, PIP dysregulation, downstream signaling alterations, and the pharmacokinetic and biodistribution properties underlying LNP *in vivo* behavior.

## 2 Lipid nanoparticle for RNA delivery: biological properties and effects on cellular systems

Having outlined the conceptual framework of L-DMD, we now examine the physicochemical, pharmacological and biological properties of LNPs that provide the mechanistic basis for this hypothesis.

### 2.1 Factors influencing nanoparticle bioactivity

Nanoparticle uptake and immune activation arise from their high surface area-to-mass ratio and reflect their integrated physical features, including size, charge, shape, and lipid composition, rather than any individual lipid^36^. Ionizable-lipid chemistry (unsaturation, branching, p*K*a), formulation conditions and manufacturing variables together influence membrane fusion, biodegradability, persistence, safety, and effectiveness^36^^,^^37^, producing emergent, process-dependent behaviors that cannot be inferred from individual components.

**Figure 2:**
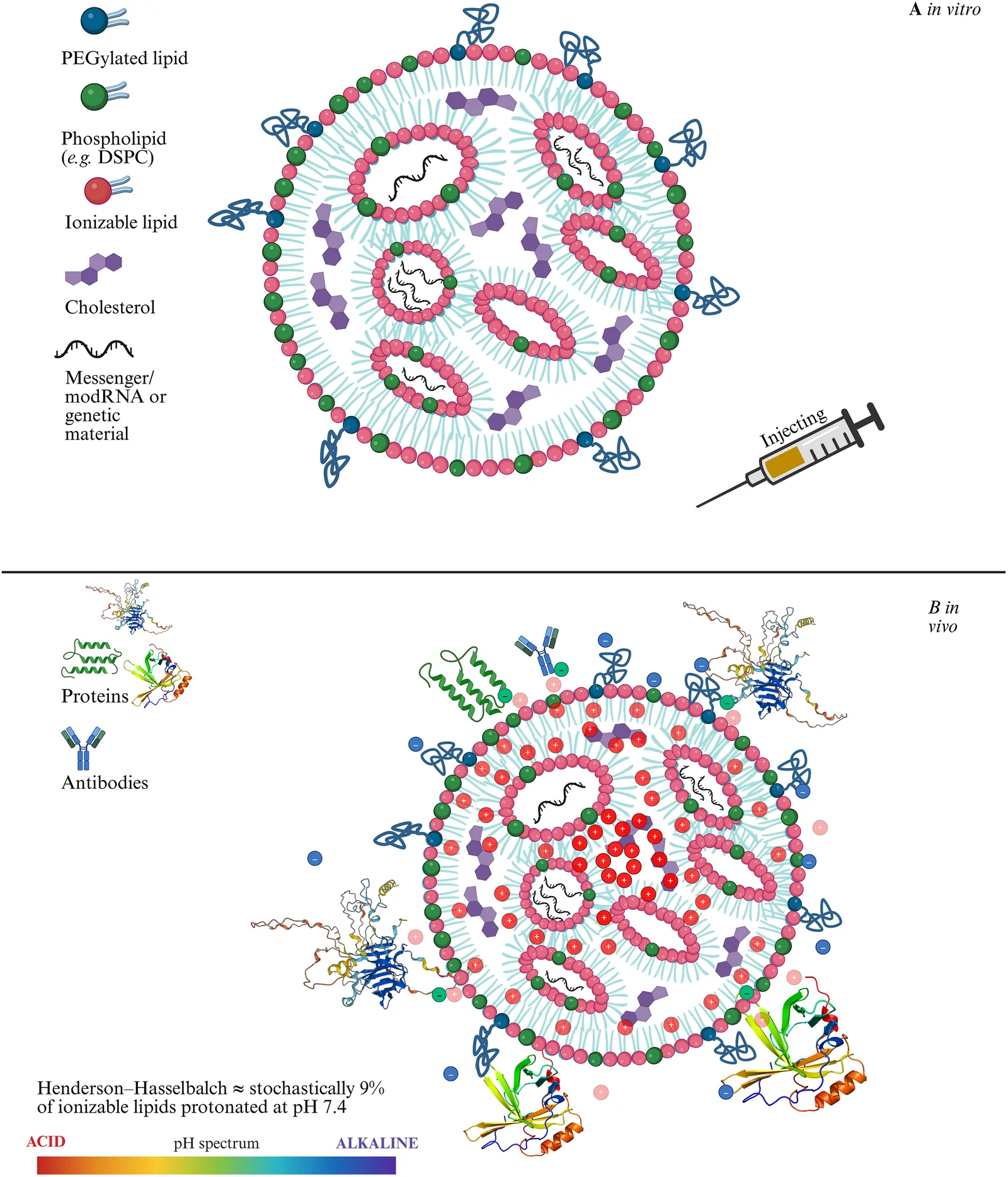
Schematic representation of the physicochemical and biological characteristics of a modRNA-loaded lipid nanoparticle (LNP). (A) Physicochemical structure of the modRNA–LNP. The lipid components self-assemble into a quasi-bilayer, forming ionizable lipid-rich domains that bind with the mRNA payload (black). The inset box shows the apparent p*K*a (∼6.2) of the ionizable lipid, resulting in about 10% protonation at neutral-to-slightly-basic pH, whether in the formulation buffer (pH 7.4 ± 0.5) or under physiological conditions (pH 7.4), thereby providing a near-neutral surface charge during circulation. A syringe icon indicates typical routes of administration (intravenous [IV] or intramuscular [IM]). (B) Protein corona formation and surface charge neutralization after plasma exposure. The same LNP after incubation with blood plasma or serum. A dense protein corona quickly forms on the LNP surface, consisting of diverse serum proteins (alpha-helices, beta-sheets, and globular structures in blue, orange, gold, and green) and immunoglobulins (Y-shaped antibodies in blue). Counterions (blue (−) symbols) on the corona interface electrostatically shield the internal positive potential, producing an apparently neutral surface (near-zero zeta potential). Red plus (+) signs inside represent residual positive electrostatic potential within the ionizable lipid-rich core that diminishes in density towards the corona, indicating charge reduction through the lipid shell and adsorbed proteins^44^. This internal-external charge asymmetry affects serum stability, biodistribution, and cellular uptake, and influences endosomal escape behavior. Created in BioRender. Seger, F. (2026). https://biorender.com/do3y6xk.

modRNA-LNP architectures are heterogeneous due to self-assembly, ranging from multilamellar vesicles with blebs to core-shell-like morphologies^8^^,^^38^. Across published platforms, estimates suggest approximately 12%^39^ to 80%^40^ of particles are empty or minimally loaded with modRNA, depending on formulation or assay used (*e.g.*, dye-binding, confocal methods) as well as batch- or manufacturing-related variability^41^. Importantly, these minimally loaded or empty LNPs (eLNPs) exhibit distinct physicochemical and functional properties^3^^,^^4^^,^^7^^,^^11^^,^ may be structurally more fluid^3^^,^^8^, and can exhibit higher per-particle fusogenicity and membrane disruption^8^. Payload content alters zeta potential, corona composition^11^^,^^12^^,^ and cellular uptake kinetics^40^^,^^42^, creating a biologically mixed exposure profile^43^ that requires human-relevant modeling to define functional response which may differ between eLNPs and loaded particles (see Section 3). Fig. 2 schematizes the physicochemical and biological characteristics of a modRNA-loaded (LNP).

### 2.2 The biocorona and biodistribution of lipid nanoparticles

Upon entry into biological fluids, LNPs rapidly acquire a dynamic coating, a biocorona, comprised of serum proteins, primarily of lipoproteins, immunoglobulins, albumin, complement, and coagulation factors that confer a new biological identity. It varies by species^45^, and is determined by both LNP characteristics (size, charge, PEG density, rigidity) and host or environmental factors^46^. In humans, it remains incompletely defined, with ApoE, vitronectin, C-reactive protein (CRP), and alpha-2-macroglobulin consistently present^47^, and it can alter LNP structure, biodistribution, and modRNA stability^48^.

LNPs remodel in plasma and lymph. Indeed, recent cryo-EM work demonstrates that LNPs do not simply adsorb proteins but rather exchange lipids and apolipoproteins with endogenous lipoproteins, producing hybrid LNP-lipoprotein assemblies resembling physiological LDL^44^. These fusions modify surface potential and fluidity, redirect uptake *via* the LDL-receptor (LDL-R) pathway and by apolipoprotein-dependent routes, and influence stability, tissue tropism, and immune recognition^49^^,^^50^.

Considering these distinctions, it is crucial to evaluate LNP distribution within the body and across cell types, as these factors affect uptake, antigen presentation, and downstream cellular processes. Biodistribution indicates where LNPs or drugs are in the body but does not itself imply cell entry or gene expression. Transfection requires the uptake and endosomal escape of nucleic acids into cells. Gene expression depends on the persistence of intact modRNA translating into protein. These processes are often conflated in studies and regulatory submissions. Recognizing this distinction is crucial for understanding toxicity, durability and efficacy, as biodistribution alone cannot predict which specific cells are transfected or the levels of protein expression.

Among the factors affecting LNP distribution, the route of administration exerts the greatest influence, more so than in small-molecule pharmacology. Factors such as syringe pressure, perfusion rate, proximity to blood and lymphatic vessels, local pH, and temperature can influence particle dispersion during intramuscular (IM) administration^51^. Following intramuscular administration, LNPs remain initially at the injection site before traversing lymphatics to draining nodes, later entering circulation primarily as lipoprotein-hybrids.

After a single intravenous infusion in rats (patisiran-like), 90% of radioactivity was found in the liver within 4 h, indicating fenestrated endothelium and ApoE/LDL-R uptake. Conversely, a single intramuscular dose of modRNA-LNP (Moderna-like) remained at the injection site for 24–48 h and only appeared in the liver after 8–48 h, indicating that the route of administration affects timing and distribution patterns, even under controlled conditions^45^.

LNPs primarily accumulate in the liver, spleen, and draining lymph nodes, which are rich in phagocytic cells of the reticuloendothelial system (RES), such as monocytes, Kupffer cells, and macrophages^52^. Such tropism toward the hepatic and lymphoid RES compartments indicates that immune activation and detoxification pathways are primary determinants of systemic LNP fate.

Recruited antigen-presenting cells (APCs) can internalize Severe Acute Respiratory Syndrome Coronavirus 2 (SARS-CoV-2) LNPs, translate the encoded spike protein, and then subsequently migrate to nearby lymph nodes, where T cell priming occurs^53^. Lesser amounts reach the heart, lungs, adrenal glands, ovaries, eyes, and other tissues *via* transcytosis or direct penetration^3^^,^^7^^,^^54^, ^55^, ^56^, ^57^, ^58^.

In humans, the ionizable lipids and the vaccine modRNA (Moderna®), quantified by mass spectrometry and quantitative polymerase chain reaction (qPCR), respectively, appeared in plasma, with T max (time to maximum observed concentration) between 4 h and 2 days, with wide inter-individual variability in timing and magnitude^59^. Buckley et al.^60^ used PET-CT in non-human primates to track rapid, stochastic LNP trafficking to lymph nodes after IM injection of a Moderna-like formulation. Quadriceps injections consistently drained to the iliac nodes with clear muscle distribution at 4 h. Deltoid injections varied, draining to axillary, apical, or pectoral nodes, with little spleen signal and no uptake in heart or other tissues (plasma kinetics not measured). These findings show that intramuscular LNP administration triggers early lymphatic trafficking and distribution prior to immune activation and clearance. Variability in *T*_max_ likely results from administration factors, tissue biology, and particle interactions with cell membranes affecting sequestration and redistribution.

Conventional radiotracing and fluorescence-based labelling approaches blur intact *vs* degraded components and lack the subcellular resolution within tissues. New peptide-tag imaging confirms the presence of intact hybrid particles in tissue^61^, highlighting the extent to which existing approaches may underestimate the complexity and heterogeneity of LNP distribution.

Uptake, or endocytosis, occurs *via* both receptor-mediated and receptor-independent mechanisms, often simultaneously, with the protein corona modulating these interactions^62^^,^^63^. Receptor-independent uptake relies on nonspecific hydrophobic and/or electrostatic forces, whereas receptor-mediated pathways, including clathrin-mediated endocytosis, depend on the local microenvironment and lipid–membrane interactions. Specific lipid components, such as the helper lipid 1,2-distearoyl-*sn*-glycero-3-phosphocholine (DSPC), can alter membrane conformation and signaling to facilitate G-protein coupled receptor (GPCR) engagement without direct binding, and lipoprotein-enriched coronas further enhance internalization^64^. Overall, the biocorona determines biodistribution, uptake and persistence, linking formulation chemistry to L-DMD risk.

### 2.3 Endosomal escape and membrane destabilization due to ionizable lipids

Endosomal escape is the rate-limiting step for RNA translation. After endocytosis, progressive endosomal acidification protonates ionizable lipids, thereby altering charge balance and triggering lipid rearrangement and membrane destabilization. Several, not mutually exclusive, mechanisms have been proposed to explain this process, including proton-driven osmotic swelling (*i.e.*, proton-sponge effect)^65^^,^^66^, promotion of non-bilayer hexagonal phases, and directed lipid mixing or pore formation facilitated by inverted-cone lipid geometries^18^. Endosomal damage, evidenced by galectin recruitment, can occur solely from ionizable lipids without cytosolic RNA delivery^67^. Smaller membrane perturbations induced by LNPs are detected and rapidly sealed by the Endosomal Sorting Complexes Required for Transport-III (ESCRT-III) machinery. Computational and experimental studies suggest that LNPs can transiently tear membranes, disrupt lipid raft organization, and tether to the endosomal membrane, enhancing escape^66^^,^^68^^,^^69^.

Escape is inefficient and remains a central bottleneck for RNA therapeutics^65^^,^^70^. Here, ‘endosomal release/escape efficiency’ refers to assay-specific estimates of the fraction of internalized LNP payload that reaches the cytosol in a functionally available form. Values are not directly comparable across assays and formulations^42^. Across representative studies, estimated endosomal release efficiencies are generally low and span roughly ∼1%–15%, as summarized in Müller et al.^42^, based on data drawn from Sabnis et al.^71^, Maugeri et al.^72^, and Gilleron et al.^73^. These efficiencies depend on the ionizable lipid structure, p*K*a, particle topology, cell type activation state, and the timing of the transient “burst” fusion window during early endosome acidification (pH < p*K*a)^42^^,^^65^^,^^74^, ^75^, ^76^. Escape occurs within minutes to a few hours after uptake, depending on formulation, cargo and assay methodology. If modRNA does not release into the cytoplasm, then maturing endosomes fuse with lysosomes, degrading modRNA and lipids. Accumulation of LNP components in lysosomes can impair function, block receptor recycling, create a cellular “traffic jam”, and prolong lipid retention, which may reduce therapy effectiveness and cause toxicity^65^^,^^76^, ^77^, ^78^, ^79^.

Indeed, LNP uptake follows a bell-shaped, nonlinear response influenced by particles and cell-specific factors. Immune cells like monocyte-derived macrophages and THP-1 cells show limited cytotoxicity, likely because their phagocytic pathways better handle LNP uptake and membrane stress^80^. Uptake and endosomal escape are further influenced by interactions between the LNP surface and surrounding biomolecules^81^^,^^82^, leading to a stochastic distribution of particle identities and protonation thresholds^76^^,^^82^. The relationship between dose and functional delivery is probabilistic, rather than linear, leading to variability in cellular responses and signaling, which are highly dose dependent^83^.

### 2.4 Spread to distant sites *via* exosomes

Once the genetic material (RNA/modRNA/DNA) escapes the endosome, it can be packaged into naturally secreted membrane-bound vesicles along with ionizable lipids and intact mRNA. Maugeri et al.^72^ showed that, in a murine model, LNPs in recycling endosomes are either expelled intact or partially degraded, thereby affecting transfection efficiency. This process involves the trafficking of a fraction of LNP-delivered mRNA together with LNP-derived lipids into intraluminal vesicles of multivesicular endosomes (MVBs), which are subsequently released *via* extracellular vesicles, thereby enabling intercellular transfer. In such models, these exosomes circulate systemically and have been shown to mediate functional RNA transfer to distant cells and enable protein translation^72^. The evidence for modRNA-LNPs transfer in humans remains preliminary and needs further investigation^84^^,^^85^.

Exocytosis serves as both a clearance route and a secondary distribution mechanism, extending modRNA or lipid fragments to the surrounding microenvironment in a paracrine manner^86^, where functional translation is possible. Importantly, recycling of endosomes, as well as eLNPs or those with blebs, may cause cellular stress, oxidative damage, and chronic inflammation^87^. These factors are not considered in biodistribution studies and may contribute to cumulative toxicity, especially with repeated doses^88^.

Spike-carrying exosomes prior to seroconversion have been detected in humans^89^, supporting the plausibility of protein relay *via* vesicular pathways, potentially extending antigen exposure and immune activation beyond initially transfected cells. However, definitive evidence for exosome-mediated transfer of vaccine-modRNA in humans remains limited and warrants further study.

### 2.5 LNP metabolism and oxidative stress mechanism

The process by which the LNPs disassemble and are metabolized is typically described as hydrolysis and clearance^90^^,^^91^. Fatty acid metabolites derived from these lipids can activate PPARs, initiating lipid-sensing and stress response pathways^78^. The long-term persistence^91^ and biodistribution^92^ have not been systematically characterized *in vivo*, particularly with respect to tissue-specific retention and potential for bioaccumulation following repeated dosing.

*In silico* studies suggest these lipids may localize within the bilayer^18^^,^^66^, further complicating predictions of LNP stability and *in vivo* behavior. Intracellularly, LNP components intercalate directly with cell membranes, triggering both repair and oxidative-stress pathways, leading to endosomal damage (detectable by galectins), immune signaling, and the production of reactive oxygen species (ROS)^93^. Oxidative stress can arise from incomplete lipid degradation, membrane perturbation, and lysosomal processing, and it is modulated by lipid structure, intracellular localization, and cellular context^67^.

Tertiary amine-based ionizable lipids can generate *N*-oxides and reactive aldehydes during manufacturing and storage, which may covalently modify modRNA, rendering it untranslatable. Packer et al.^94^ first identified such adducts in 2021. They can be mitigated by buffering or novel lipid designs^95^. These adducts may generate aberrant molecules that activate interferon-driven innate immune pathways^96^^,^^97^ or potentially modify cellular proteins through electrophilic mechanisms. Ionizable lipids commonly incorporate unsaturated acyl chains susceptible to peroxidation, producing electrophilic aldehydes such as 4-hydroxynonenal (4-HNE), a well-established oxidative-stress product known to disrupt protein folding and lysosomal function^78^^,^^98^. However, direct evidence that modRNA-LNP administration generates 4-HNE at biologically significant levels in humans is currently lacking, and any clinical impact remains speculative.

Nonetheless, LNP metabolism may be viewed not only as producing degradative metabolites but also as a potential source of reactive, bioactive lipid intermediates that contribute to sustained inflammatory or stress signaling^90^^,^^91^.

### 2.6 Activation of the immune system

LNPs can activate the immune system independently of spike protein expression. Within the adaptive immune system, two mechanisms have been identified. First, IgE-mediated anaphylaxis arises from antibodies induced against PEGylated particles in the biocorona^3^^,^^99^^,^^100^. Such reactions are rare but potentially life-threatening. Second, exposure to PEGylated LNPs can induce anti-PEG IgM antibodies, leading to the accelerated blood clearance (ABC) phenomenon, in which subsequent doses are rapidly cleared, reducing efficacy and altering exposure profiles^101^.

Third, LNPs also activate innate immunity through complement- and receptor-mediated pathways. Complement activation-related pseudo-allergy (CARPA) represents a blood-facing innate immune reaction to nanoparticles, including PEGylated drugs^102^^,^^103^. CARPA is mediated by complement activation and anaphylatoxins (Complement (C)3a, C5a), often triggered by anti-PEG IgM antibodies, which can activate mast cells and release histamine, leading to acute infusion reactions^103^.

Evaluation of CARPA typically relies on porcine and non-human primate models that recapitulate human cardiopulmonary responses; to date, to our knowledge, only one published study^104^ has directly examined modRNA-LNP administration in a porcine CARPA model using intravenous administration, confirming complement activation and CARPA-like hemodynamics. Allergic reactions observed after modRNA COVID-19 vaccination may be mediated by this phenomenon^105^ and have prompted calls for alternative PEG excipients to mitigate CARPA^106^. Clinically, patisiran (an siRNA-LNP therapeutic for the treatment of hereditary transthyretin-mediated amyloidosis, Onpattro®) infusions can also trigger CARPA, sometimes severely, through complement activation^107^^,^^108^.

LNPs can activate the innate immune system by transfecting immune cells, providing an intrinsic adjuvant-like activity^79^ that aids in antibody production; LNPs can activate TLR3, 7, 8, and 9 in the endosome^109^. Additionally, LNPs bind to diverse cell membrane receptors such as CRP, TLR4, and TLR2, and can activate the NOD-Like Receptor Protein 3 (NLRP3) inflammasome^110^. These immune effects highlight the need to distinguish among biodistribution, transfection, and gene expression, as they are context-dependent and may cause pathogen-like effects beyond just cytotoxicity.

### 2.7 Delivery architecture as a determinant of membrane stress and possible systemic risk

The biological effects of nucleic acid therapeutics are determined not solely by RNA modality, but also by delivery architecture and membrane interactions. Comparative analysis of approved platforms reveals a continuum of membrane engagement and persistence not captured by conventional pharmacokinetic or biodistribution frameworks.

This principle is evident when examining clinically approved RNA therapeutics. The siRNA therapeutic inclisiran (Leqvio®) achieves durable LDL-cholesterol lowering through triantennary *N*-acetylgalactosamine (GalNAc)-mediated receptor-targeted delivery to hepatocytes, and has been evaluated in large, repeated-dose clinical trials with extended follow-up^111^^,^^112^. Its favorable safety profile, supported by precise cellular targeting, low administered doses, and infrequent dosing, mitigates systemic exposure and inflammatory risk, suggesting that the delivery strategy, not the RNA modality itself, may be a key determinant of long-term tolerability. Patisiran (Onpattro®) has a safety profile that includes transient elevations in transaminase levels *(*often resolved with dose adjustment) and infusion reactions, but little evidence of progressive hepatotoxicity over 18 months of treatment^107^^,^^113^. This contrasts with the acute reactogenicity observed with modRNA-LNP vaccines, although direct comparisons are confounded by route (IV *vs.* IM) and dosing frequency.

In contrast, the European Medicines Agency (EMA)'s assessment of the self-amplifying-RNA COVID-19 vaccine zapomeran (Kostaive®), which employs LUNAR (ATX-126) ionizable lipids designed for improved degradability^114^, reported very slow clearance of ATX-126 with tissue half-life not determinable for most organs, and an estimated half-life in muscle of 31-64 days^115^. Collectively, these products define a continuum of RNA delivery: from targeted, non-particle delivery (Leqvio®) to hepatotrophic siRNA-LNP delivery (Onpattro®), and to lipid-based vaccine platforms employing ionizable lipids tuned for increased endosomal fusion and cytosolic release (Kostaive®) (Table 1^19^^,^^53^^,^^107^^,^^111^, ^112^, ^113^, ^114^^,^^116^^,^^117^). This is consistent with experimental structure–activity analyses showing that lipid architecture and ionization state influence bilayer disruption and toxicity^19^.

**Table 1:** Comparative pharmacology and safety profile of approved RNA nanoparticle therapeutics.

Platform (example)	Route/vehicle	Primary delivery mechanism	Approximate endosomal escape^a^	Membrane activity^b^	Potential membrane risk^c^	Common safety signals	Ref.
GalNAc–siRNA (inclisiran)	SC, Ligand–siRNA conjugate, No LNP.	Hepatocyte-targeted receptor uptake (ASGPR).	Sufficient at low doses (No LNP).	Minimal: Negligible disruptive capacity.	√ (No ionizable lipids).	Primarily injection site only.	Di Fusco et al.^111^ Wright et al.^112^
siRNA–LNP (patisiran)	i.v., Ionizable lipid, (DLin-MC3-DMA).	Hepatic fenestration + ApoE mediated LDL-R.	∼1% (model-dependent).	Modest; Transient endosomal lesions repaired by ESCRT.	√√ (Hepatotropic, low p*K*_a_ 6.4).	↑Transaminases; ↓ Vitamin A levels; Infusion reactions (CARPA).	EMA Onpattro EPAR 2018.^107^ Urits et al.^113^
modRNA–LNP (SARS-CoV-2 vaccines)	i.m., Ionizable lipid, (ALC-0315/SM-102).	Immune activation + lymphatic drainage + systemic distribution.	≤∼15% (context-dependent).	High; Sustained endosomal/lysosomal engagement.	√√√ (systemic distribution, p*K*_a_ 6.1–6.7).	Systemic reactogenicity (fever myalgias), Rare myocarditis, Rare anaphylaxis.	EMA EPAR Comirnaty 2021.^53^ EMA EPAR Moderna 2021^116^. Wong et al. ^117^
saRNA–LNP (zapomeran/SARS-CoV-2 vaccine)	i.m., Ionizable lipid, (ATX-126/LUNAR lipids).	Prolonged RNA replication + front-loaded lipid-mediated delivery.	Variable; amplification design.	High; ionizable lipid with saRNA prolonging innate activation.	√√√ (very slow clearance, p*K*_a_ undisclosed/proprietary).	Systemic reactogenicity ≈ modRNA-LNPs.	Atmuri et al.^19^ EMA EPAR Kostaive^114^

√, minimal, √√√, highest relative risk category. This framework requires prospective validation.

ApoE, apolipoprotein E; ASGPR, asialoglycoprotein receptor; CARPA, complement activation-related pseudoallergy; ESCRT, endosomal sorting complexes required for transport; GalNAc =*N*-acetylgalactosamine; i.m., intramuscular; i.v., intravenous; LDL-R, low-density lipoprotein receptor; L-DMD, lipid-driven membrane dysfunction; LNP, lipid nanoparticle; LUNAR, Lipid-enabled and Unlocked Nucleomonomer Agent modified RNA; modRNA, nucleotide-modified RNA; p*K*a, acid dissociation constant; SARS-CoV-2, severe acute respiratory syndrome coronavirus 2; SC, subcutaneous; saRNA, self-amplifying RNA; siRNA, small interfering RNA.

a Endosomal escape varies by cell type, ionizable lipid p*K*a, and assay method (*e*.*g*., fluorescence dequenching, galectin recruitment). Values represent estimates from *in vitro* and animal models; human data are limited.

b Membrane activity represents qualitative assessment from regulatory filings and published mechanistic studies and does not imply difference in lipid persistence or systemic half-life.

c Potential L-DMD risk: based on ionizable lipid p*K*a, membrane fusogenicity, and biodistribution.

These data indicate that ionizable LNPs cannot be regarded as biologically inert carriers whose effects end once RNA is released. They function as supramolecular, membrane-active assemblies that persist within endosomal and lysosomal membranes, engaging repair circuits, oxidative stress pathways, and innate immune signaling. These processes are highly context-dependent, vary across tissues and individual cells^118^, and are not fully captured by conventional biodistribution or transfection metrics. Together, the continuum of observations supports the lipid-driven membrane dysfunction (L-DMD) hypothesis as a coherent explanatory framework for the durable, tissue-specific and heterogeneous outcomes associated with modRNA-LNP exposure. These mechanistic insights give rise to the core L-DMD hypothesis: membrane- and vesicle-level perturbations may represent the missing link that couples delivery architecture to cellular dysregulation.

Building on these considerations, the following section investigates how the biological disposition, pharmacodynamic, and physicochemical properties of modRNA-LNP systems may produce detectable molecular signatures *in vivo*. If membrane-associated uptake, intracellular trafficking, endosomal escape, or LNP restructuring perturb local signaling environments, such effects are expected to produce coordinated, system-level transcriptomic and proteomic shifts rather than isolated pathway activation. Omics-based profiling therefore provides a suitable framework to evaluate whether modRNA-LNP exposure is associated with broad molecular changes consistent with membrane-centered dysregulation.

## 3 Omics data indicating membrane dysfunction secondary to LNP transfection

Omics approaches, including transcriptomics, metabolomics, lipidomics, and proteomics, are exploratory tools for analyzing cellular and organismal responses in experimental systems. They generate high-dimensional datasets that reveal molecular patterns and pathway alterations, providing reproducible, falsifiable signatures that support hypothesis generation rather than causal proof. In the context of modRNA-LNP COVID-19 vaccines, these datasets enable systematic assessment of intracellular responses to both eLNPs and payloaded formulations following uptake, endosomal processing, and translation of modRNA. Although primarily descriptive, omics data provide a foundation for mechanistic interpretation, including evaluation of membrane-associated dysfunction. A key limitation is that transcriptomic changes alone cannot establish functional pathway activation without proteomic validation, while proteomic data alone cannot fully reconstruct upstream regulatory or membrane-level processes.

To date, omics analyses of LNPs remain limited, particularly for longitudinal human comparisons between vaccinated and unvaccinated cohorts. Transcriptomic, lipidomic, and proteomic datasets are therefore of primary interest, as they provide the most direct insights into altered signaling and protein activity states following LNP exposure. Established signaling pathway reconstructions, especially from cancer and immunologic research, provide a validated framework for interpreting pathway crosstalk and network dynamics and guide the hypothesis-driven part of this analysis. Accordingly, we organized this section into three parts: (1) omics data from eLNP-treated mouse models, (2) available human omics datasets, and (3) evaluation of whether convergent transcriptomic and proteomic signatures support the L-DMD hypothesis, while clearly distinguishing exploratory associations from mechanistic inference. Later sections integrate these findings across models and interpret them within the L-DMD framework.

### 3.1 Mouse data

In 2021, Ndeupen et al.^119^ published a pioneering *in vivo* study, in which they analyzed how a biological system responds to eLNPs. Their pharmacokinetic investigation, in which wild-type (WT) C57BL/6 (B6) mice were injected intramuscularly with 10 μg of eLNPs, revealed thousands of changes in gene expression. The chosen dosage corresponds to the typical design for pharmacodynamic evaluation^120^^,^^121^. Furthermore, these changes were also evident in the Luminex panel at the protein level.

Regarding the number of up- or down-regulated genes, the study analysis revealed that “*with P < 0.05 and FDR [False Discovery Rate] < 0.05, 9508 genes and 8883 genes, respectively, were differentially expressed*.” A marked upregulation of genes involved in monocyte and granulocyte development, recruitment, and function (*e.g.*, *Ccl2*, *Ccl3*, *Ccl4*, *Ccl7*) was observed. The Kyoto encyclopedia of genes and genomes (KEGG) transcriptomic gene set enrichment analysis (GSEA) revealed a pronounced induction of inflammatory cytokines, including Interleukin-1 (IL-1) beta (*β*), granulocyte–macrophage colony-stimulating factor, and IL-6 signaling pathways, which are hallmark markers of acute innate and adaptive immune activation^122^, ^123^, ^124^, ^125^, ^126^.

NF-*κ*B was upregulated by an estimated 2.5–2.6 log_2_-fold. In contrast, the TCA cycle and PPAR signaling pathways were markedly downregulated, with an approximately 2.0-log_2_ fold reduction in expression. The upregulation of genes associated with TLRs, NOD-like receptors, and RIG I-like receptors suggests a robust system-wide immunological response^127^. Hematopoietic cell lines, in particular, showed a more than 2 log_2_-fold increase in gene expression, indicating pronounced activation within stem and progenitor compartments that may be driven by the combined signaling of TLRs^128^, NOD receptors^129^, and RIG I^130^ pathways and/or the internalization pathways of the LNPs. These findings are consistent with the single-cell-based flow cytometric experiment by Parhiz et al.^131^.

Furthermore, the observations of Ndeupen et al.^119^ are consistent with the *in vitro* experiment made by Zelkoski et al.^132^. in human monocytes, showing that eLNPs trigger TLR4-dependent signaling that bifurcates into a strong myeloid differentiation factor 88 (Myd88)/NF-*κ*B response and a parallel, though weaker, TIR-domain-containing adapter-inducing interferon-*β* (TRIF)-mediated interferon regulatory factor (IRF) activation. Their enzyme-linked immunosorbent assay and transcription analysis revealed that Jun, a component of the AP-1 transcription factor complex, activated *via* the c-Jun N-terminal kinase (JNK)-MAPK cascade downstream of TLR4/MyD88 signaling, exhibited a 1.93-fold log_2_ expression change, whereas the JAK–STAT axis, typically associated with interferon signaling^133^, showed a more modest 1.13-fold log_2_ change.

Korzun et al.^134^ investigated the reactogenicity of eLNPs compared with Luciferase (Luc) mRNA-LNPs *in vivo,* both as a single dose and after multiple doses, in C57BL/6 (wild-type) mice and in MyD88, TLR4, and TRIF-knockout lines. The LNPs were produced microfluidically (Onpattro®-like, Pfizer®-like, Moderna®-like) and injected intraperitoneally. The ionizable lipids were the only variable changed. Reactogenicity was quantified by sickness behavior (reduced food intake and weight loss). TLR4 dependence was tested by pharmacological inhibition with resatorvid (TAK-242) (3 mg/kg) given 2 h prior to each dose in the eLNPs groups. Gene expression was analyzed using the NanoString Counter Mouse Inflammation Panel (liver and hypothalamus tissue) and qRT-PCR, supplemented by Enzyme-Linked Immunosorbent Assay (IL-6, IL-1*β*, TNF-*α*), Lipocalin 2, and Fluorescence-Activated Cell Sorting analysis of peritoneally obtained immune cells.

The authors showed that TLR4 and MyD88, in particular, are necessary for the initiation of these reactions and that pharmacological inhibition of TLR4 (*e.g.*, with TAK-242) can attenuate the reactogenicity of eLNPs, and thus also the disease-related behavioral changes. These findings underscore the importance of the TLR4/MyD88 axis not only for proinflammatory gene expression, but also for physiological behavioral responses associated with sickness behavior. This finding is consistent with *in vitro* experiments performed by Zelkoski et al.^132^.

Similarly, Korzun et al.^134^ described an inflammatory signature for ionizable lipids, including induction of the proinflammatory cytokine genes *Il1b* and *Il6*, as well as the chemokine genes *Ccl2*, *Ccl4*, *Cxcl2*, and *Cxcl10*, findings consistent with Ndeupen et al.^119^ and indicative of enhanced inflammatory gene expression and downstream pathway activation.

Further examination of the raw transcriptional data provides additional support for the observations of Ndeupen et al.^119^. Specifically, analyses of whole-tissue expression reveal substantial regulation of key signaling nodes, including Ras homolog gene family member A (*Rhoa*), phosphatidylinositol-4-phosphate 3-kinase catalytic subunit type 2 gamma (*Pik3c2g*), Rho-associated coiled-coil containing protein kinase 2 *(Rock2*), *Mapk3* (also known as extracellular signal-regulated kinase 1 (*Erk1*), and *Stat3*. These changes, observed in whole tissue, indicate a systemic activation of RAS/PI3K/ROCK/ERK signaling pathways that likely involves multiple cell types, including immune and epithelial cells. Analyses restricted to isolated immune cell subsets may not fully capture these effects^133^, ^134^, ^135^, ^136^. Canonical genes such as Kirsten Rat Sarkoma (*Kras*) were not significantly altered in immune-cell-enriched subsets, whereas downstream and parallel pathways show robust modulation, highlighting the importance of considering global transcriptional responses to eLNPs.

Another study supporting these observations published by Luo et al.^137^ investigated proteomic alterations induced by eLNPs lacking a modRNA payload. Depending on the formulation, the various nanocarriers were administered intranasally, intramuscularly, intravenously, orally, or intradermally, with doses ranging from 0.0005 mg/kg to 0.5 mg/kg (LNPs, liposomes, polyethylenimine complexes) or 50.725 mg/kg (DNA origami) and 10^13^ GC/mL adeno-associated viruses.

The authors compared payload-containing and no-payload LNPs with phosphate-buffered saline [PBS] controls. They detected 375 differentially expressed proteins, of which 240 were upregulated and 135 downregulated in the no-payload LNP group *in vivo*. As determined by Reactome pathway analysis, these changes were linked to metabolic processes, particularly ribosome function, protein translation, and RNA metabolism. Specific markers, including Ribosomal Protein 2, 6, 11, 15, Eukaryotic Initiation Factor 4B, 2B3 were found to be differentially regulated.

These data, derived from *in vivo* Omics analyses, provide early insights into the broad immunological and signaling effects elicited by LNPs.

### 3.2 Human data

To the best of our knowledge, there are only two existing *ex vivo* human studies providing direct molecular readouts following COVID-19 modRNA-LNP vaccination: Knabl et al.^138^ (concerning *ex vivo* buffy coat transcriptomics) and Hickey et al.^139^ (concerning longitudinal serum proteomics). Other studies are cited solely to provide context.

Knabl et al.^138^ conducted an *ex vivo* transcriptomic study to assess the systemic effects of modRNA packaged in LNPs in both elderly individuals with comorbidities and healthy younger vaccinated individuals.

The first study group consisted of nine hospitalized Beta-variant COVID-19 patients, of whom four had received the first dose of BNT162b2 8-11 days before symptom onset, while five remained unvaccinated; all vaccinated patients and three of the five unvaccinated received dexamethasone. Immune transcriptomes (MSigDB-GSEA) were analyzed at days 7–13, 20–32, and 42–60 after symptom onset. In the raw data, thousands of genes in the transcriptomics were statistically significantly changed (up- or downregulated) after the BNT162b2-application. As reported by the authors, COVID-19 symptoms (Beta variant) developed in all patients, including those infected shortly after the first BNT162b2 dose^138^. MSigDB-GSEA analysis of the four vaccinated patients revealed acute changes in gene expression across thousands of genes. One elderly comorbid patient died before the end of the study. Signaling pathways affected included JAK–STAT3, mTORC1, and KRAS, among others, with many transcriptomic changes reaching statistical significance.

The canonical ERK–MAPK signaling pathway involves several key proteins: rat sarcoma (RAS), rapidly accelerated fibrosarcoma (RAF), mitogen-activated protein kinase (MEK), and extracellular signal-regulated kinase (ERK), and it has extensive crosstalk with the mTORC1/2-pathways through feed-forward and feed-back mechanisms^140^.

Notably, *TNFA*, *via* NF-*κ*B signaling, was upregulated in both study groups (see next paragraphs), similar to the activation observed by Zelkoski et al.^132^ and Ndeupen et al.^119^. It remains unclear whether this reflects canonical (*RELA*/p65–*IKB*-dependent) or non-canonical (*NIK*–*RELB*-mediated) NF-*κ*B activation^141^.

Moreover, Knabl et al.^138^ detected significant mTORC1 signaling across doses 1 to 3, with 10 overlapping genes reaching statistical significance, underscoring that both mTORC1 and p53 are co-activated under physiological and genotoxic stress. KRAS signaling can engage NF-*κ*B through multiple downstream routes (including RAF/MEK/ERK and PI3K/AKT branches)^142^, thereby promoting transcriptional programs that support cell survival and stress adaptation. Distinguishing canonical from non-canonical NF-*κ*B activation in these samples requires targeted inspection of pathway components (*e.g.*, *RELA*/p65 phosphorylation and *IKB* degradation for the canonical arm; *NIK*/*RELB* dynamics for the non-canonical arm) at the transcript and protein level^142^. Moreover, the four elderly patients showed significant upregulation of the JAK-STAT5 pathway with *IL-2* and the JAK–STAT3 pathway with *IL6*.

The authors later included eight healthy, naive individuals receiving their first vaccine dose and analyzed transcriptional changes 7–10 days post-vaccination. In these naive individuals, selective upregulation of the transcription factor *E2F8* and specific interferon-stimulated genes was observed *via* p53-regulated pathways, while *E2F1* and cyclin A1 (*CCNA1*) remained largely unchanged. Similarly, only three canonical mTORC1 target genes (cell division cycle 25A (*CDC25A*), ribonucleotide reductase family member 2 (*RRM2*), and BUB1 mitotic checkpoint kinase (*BUB1*)) were modulated, indicating that global mTORC1 activation was minimal yet statistically significant at this early time point. *CDC25A*, *RRM2*, and *BUB1* mark the transitions G1/S (*CDC25A*), DNA synthesis (*RRM2*), and mitosis (*BUB1*)^143^.

Taken together, these findings indicate that the observed mTORC1-related transcriptional patterns are unlikely to be primarily driven by dexamethasone treatment, particularly given its half-life of 36–54 h^144^, which is unlikely to account for weeks-long pathway activation. If corticosteroid exposure were the dominant driver, suppression rather than persistence of mTORC1-associated transcriptional programs would be expected.

Hickey et al.^139^ conducted one of the first proteomic analyses examining molecular alterations following BNT162b2 and mRNA-1273 vaccination. Serum samples from adults vaccinated with BNT162b2 or mRNA-1273 were analyzed longitudinally for proteomic changes. Statistical analyses, including predictive modeling, identified key markers and signaling pathways. One month post-third dose, both modRNA vaccines modulated pathways linked to RAS/MAPK and ubiquitin-mediated protein regulation, with consistent upregulation of UB2D1/PolyUbiquitin K48, indicating shared activation of protein degradation. Phosphoinositide-dependent signaling, including PI3K–PI(3,4,5)P_3_–AKT, PIP_2_-dependent calcium mobilization, and PIP-regulated endocytic trafficking, was modulated, reflecting early membrane–proximal post-translational signaling. Downstream effects varied by vaccine platform and gender.

ChaC glutathione-specific gamma-glutamylcyclotransferase 1 and insulin signaling upregulation diverged between BNT162b2 and mRNA-1273, suggesting platform-specific LNP/ionizable lipid effects on stress and metabolic signaling. A transient induction of cytochrome P450 phase I metabolic pathways occurred exclusively in male BNT162b2 recipients, without activation of the constitutive androstane receptor (CAR)/pregnane X receptor (PXR), arguing against xenobiotic induction and pointing to sex-specific or context-dependent hepatic modulation. In the long term, six-month assessments show persistent activation of translational and ribosomal pathways, with male mRNA-1273 recipients exhibiting additional cap-dependent translation initiation and AKT signaling.

### 3.3 Convergent findings across studies and platforms

Despite differences in model systems (mouse *versus* human), omics platforms (transcriptomics *versus* proteomics), LNP formulations (MC3, ALC-0315, SM-102), and experimental designs, several molecular signatures appear consistently across studies.

(1) Multiple lines of evidence indicate modulation of phosphoinositide-related pathways. These include downregulation of *PIK3C2G* (class II PI3K) across independent mouse datasets, phosphoinositide-dependent signaling alterations observed in human serum proteomics, and dysregulation of ESCRT-associated pathways involved in membrane repair and endocytic trafficking.
(2) Inflammatory and stress-associated signaling pathways are reproducibly engaged, including NF-*κ*B activation, upregulation of TNF-*α*, IL-6, and IL-1*β*, chemokine induction, and evidence of NLRP3 inflammasome involvement (for proteomics and transcriptomics across species). TLR4/MyD88-dependent signaling is consistently implicated, with indications of pathway bias, depending on cellular and experimental context.
(3) Metabolic and detoxification pathways are affected in a context-dependent manner. These include downregulation of cytochrome P450-associated xenobiotic metabolism, suppression of PPAR and AMPK signaling pathways, and attenuation of TCA cycle activity, consistent with altered lipid and energy homeostasis.
(4) Multiple signaling cascades downstream of membrane–proximal events are activated, including RAS/MAPK, PI3K/AKT/mTOR, and JAK–STAT pathways, as observed in both mouse and human datasets.

Importantly, several of these molecular perturbations are observed following exposure to empty LNPs lacking RNA payload, indicating that components alone are sufficient to induce broad transcriptomic and proteomic responses. The convergence of phosphoinositide-dependent signaling changes, PI3K modulation, engagement of the membrane repair pathway, and downstream signaling activation provides empirical support for the L-DMD hypothesis, which posits membrane-level dysregulation as a central initiating event.

However, the biggest hurdle is distinguishing between payloaded LNPs and eLNPs due to their altered *in vivo* behavior, as outlined in Section 2. In addition, antagonistic and/or synergistic effects are expected during endosomal escape, subsequent payload release, and transcription. This was demonstrated by Luo et al.^137^ in their proteomics analysis comparing empty *versus* payloaded LNPs.

Taken together, the observed disparities in LNP behavior suggest a shift from empirical description toward a systems-level mechanistic interpretation.

## 4 Proposed mechanistic hypothesis derived from the omics data: L-DMD as a central node

In this section, we present and discuss several pathways at the level of mechanistic signaling nodes, with direct experimental support (derived from transcriptomic and proteomic data). This serves as a reference framework for network dynamics, rather than as a complete causal chain or disease model. The previous omics signatures will be interpreted through three types of evidence: 1) Direct observations (*e.g.*, PIK3C2G downregulation), 2) Mechanistic nodes with cross-study validation (*e.g.*, TLR4/MyD88), and 3) System-level implications requiring prospective studies (*e.g.*, cell-cycle dysregulation). Phosphoinositides (PIPs) are emphasized as central organizers of these pathways, providing membrane–proximal coordination of signaling nodes and network integration.

### 4.1 Disruption of the ESCRT circuit and phosphatidylinositol signaling

The most striking observation from the proteomics study by Hickey et al.^139^ concerns a reduction in endocytic activity and a downregulation of the ESCRT-circuit, both of which could indicate a disruption of the PI cycle (which refers to the conversion and recycling of PIPs)^145^, ^146^, ^147^. It has been shown that the modRNA-LNPs induce endosomal membrane ruptures^88^, which are detected by the ESCRT machinery. Small disruptions are quickly sealed by the ESCRT-circuit^148^ in crosstalk with PIPs before disruption accelerates, and larger perturbations trigger NLRP3 and binding by galectins to signal repair. Interestingly, Förster III et al.^149^ demonstrated that mRNA-carrying LNPs can also induce lysosomal rupture, which likewise activates the NLRP3 inflammasome and reduces mRNA translation efficiency.

This is consistent with the review by Hurley et al.^150^, which summarizes ESCRTs as central to cell membrane repair mechanisms and, beyond that, to the organelle transport system. ESCRT complexes are recruited to membrane domains in part through interactions with phosphoinositide pools that define membrane identity, and their function in sorting ubiquitinated payload integrates ubiquitin signaling with membrane remodeling^148^. Distinct ESCRT subunits contain ubiquitin-binding motifs that recognize ubiquitylated membrane proteins and phosphoinositides, allowing coordinated recruitment and assembly of ESCRTs at phosphoinositide-rich membranes, thereby linking ubiquitin-driven payload recognition to membrane deformation and trafficking. In summary, this pathway identified by proteomics provides supporting evidence for our hypothesis.

### 4.2 Downregulated xenobiotic metabolism by cytochrome P450 enzymes

A notably downregulated KEGG pathway identified by Ndeupen et al.^119^ and sex-specific for BNT162b2 in Hickey et al.^139^ was the metabolism of xenobiotics by cytochrome P450 (CYP) enzymes across species. Available data also imply perturbations in hepatic xenobiotic metabolism, warranting closer examination of CYP enzymes as membrane-embedded pharmacologic sensors. Biochemical and structural studies demonstrate that these enzymes are functionally embedded in biological membranes^151^, ^152^, ^153^.

Rises in pro-inflammatory cytokines, including IL-6, TNF-*α*, IFN-*γ*, IL-2, IL-1*α*, and IL-1*β* further suppress hepatic CYP1A2, CYP2C9, CYP2C19, and CYP3A4^154^^,^^155^. Such reductions usually only occur after marked systemic inflammation (*e.g.*, sepsis, CRP >100 mg/L)^156^. Clinically, case reports and cohort analyses document relevant changes in clozapine pharmacokinetics post-vaccination, in some cases leading to neutropenia and hospitalization^157^, ^158^, ^159^.

The mechanism is consistent with inflammation-mediated suppression of CYP enzymes, particularly CYP1A2 and CYP3A4, which are central to clozapine metabolism^160^. Comparable effects have been reported for other CYP-metabolized drugs, including statins, benzodiazepines, antiepileptics, and immunosuppressants, which are predominantly CYP3A4^161^ or CYP2C9 substrates^162^, likely reinforced by altered membrane conditions. Together, these findings identify context-dependent modulation of CYP-associated pathways following modRNA-LNP vaccination, prompting mechanistic exploration of membrane-related processes.

CYP enzymes are essential for xenobiotic detoxification and endogenous lipid metabolism. They perform crucial biological functions, including cholesterol and fatty acid metabolism, vitamin D activation, and the synthesis of prostacyclins and thromboxanes from arachidonic acid^163^.

ModRNA-LNPs can transiently remodel local cellular and vesicular membranes by altering phospholipid organization and membrane curvature through fusogenic and/or receptor-binding interactions^164^. Ionizable lipids with specific amine headgroups have been reported to directly interact with membrane immune receptors such as TLR4 and Cluster of Differentiation 1d, supporting the possibility that LNP components can perturb native lipid microdomains that normally stabilize receptor complexes and membrane-bound enzymes^110^.

The exact mechanisms are still under intense investigation. However, such microdomain disruption provides a plausible mechanistic basis for the ‘decoupled’ TLR4 signaling patterns^165^ observed in murine studies by Korzun et al.^134^ and Ndeupen et al.^119^, as well as in luminescence assays by Zelkoski et al.^132^, and could additionally contribute to CYP activity at the hepatocellular membrane interface^166^. In addition, independent biophysical studies have further demonstrated rapid pH-responsive structural transformations of ionizable LNPs that induce curvature stress and lipid packing rearrangements consistent with membrane remodeling behavior^74^^,^^167^^.^

Furthermore, CYP enzymes are functionally embedded in biological membranes, with their binding, orientation activity depending crucially on the lipid composition: Anionic phospholipids such as phosphatidylserine or phosphatidylglycerol mediate stable docking of positively charged CYP domains *via* electrostatic interactions^168^, while phosphatidylethanolamine, due to its small head group, induces membrane curvature and loosening of the packing, thus enabling partial insertion and correct orientation of the enzyme; this lipid-dependent embedding is dynamic, influences the opening of membrane-side substrate channels and coupling to redox partners^169^, and thus directly determines catalytic efficiency, whereby this principle is not limited to ER-localised CYPs, but also applies to mitochondrial steroidogenic CYP enzymes^170^. Therefore, perturbations to membrane composition would be expected to directly alter CYP and catalytic performance.

In summary, CYP downregulation likely reflects both cytokine-mediated transcriptional suppression and direct perturbation of membrane microdomains by ionizable lipids, consistent with the L-DMD prediction that membrane-embedded enzyme function is sensitive to lipid reorganization. Corroborating these molecular events, clinical evidence of post-vaccination hypercholesteremia^171^ supports the concept that impaired hepatic detoxification and membrane-associated enzyme modulation translate into measurable serum lipid changes at the population level.

### 4.3 Are the TLR4 reactions biased? What the mouse data reveal

In line with the CYP observations, the results by Ndeupen et al.^119^, Korzun et al.^134^, and Zelkoski et al.^132^ indicate a biased TLR4 dual-pathway activation pattern, with evidence pointing toward a stronger MyD88-dependent response relative to TRIF-dependent signaling in certain contexts. These observations raise the possibility of divergent or potentially decoupled TLR4 signaling. Such a bias may originate upstream at the cell membrane level and could be linked to early membrane dysfunction, consistent with patterns described by Zelkoski et al.^132^ and related literature on ligand-specific TLR4 modulation^172^, ^173^, ^174^.

Building on considerations of membrane structure and lipid microdomain organization, it is mechanistically plausible that early plasma membrane alterations could perturb TLR4 receptor localization, orientation, and/or associated phosphoinositide dynamics (*e.g.*, PI(4,5)P_2_). This could potentially contribute to biased or divergent dual-pathway activation. As a working hypothesis, this concept may connect our proposed membrane-level dysregulations to the observed TLR4 signaling patterns, given the established interconnection of TLR4 with PI(4,5)P_2_^172^, ^173^, ^174^.

modRNA N1-methylpseudouridine (m1*ψ*) modifications suppress endosomal TLR recognition^175^, but ionizable lipidamine head groups are capable of simultaneously activating membrane-associated TLR4 and Cluster of Differentiation 1d^110^, which could lead to a paradoxical state of simultaneous TLR suppression and activation. This should also be considered in omics data. This dual, potentially opposing regulation may contribute to complex or seemingly contradictory immune activation signatures and should therefore be carefully considered when interpreting omics datasets.

Within the context of the L-DMD hypothesis, such a mechanism provides a plausible explanation for divergent TLR4 signaling patterns and supports the concept that supramolecular LNP properties can modulate receptor signaling independently of the RNA payload. While direct experimental evidence linking LNP-induced membrane perturbation to TLR4 pathway bias remains limited, extensive literature demonstrates that TLR4 signaling is highly sensitive to lipid raft integrity and local PI(4,5)P_2_ availability, thereby making this a testable mechanistic link within the L-DMD framework^176^^,^^177^.

### 4.4 Upregulation of multiple inflammatory markers

Ndeupen et al.^119^ showed a remarkable cytokine and chemokine profile. The simultaneous upregulation of TNF-*α* IL-1*β*, and IL-17 signaling pathways is notable, since these cytokines form a pro-inflammatory axis frequently implicated in the pathogenesis of autoimmune and chronic inflammatory conditions^178^.

While IL-6 can modulate autophagy in the context of oxidative stress^179^, and IL-1*β* levels may be influenced by autophagic clearance of pro-IL-1*β*^180^, their elevation in this setting more likely reflects a broader integration of NLRP3-inflammasome activation^179^, NF-*κ*B signaling, and MAPK pathway dysregulation^181^, ^182^, ^183^. Similarly, *β*-chemokines such as CCL2, CCL3, CCL4, and CCL7 contribute not only to immune cell recruitment^184^^,^^185^, but may also intersect with autophagy-related transcriptional regulation (*e.g.*, *via* FOXK1)^186^^,^^187^. Korzun et al.^134^ observed comparable patterns.

Taken together, the coordinated upregulation of IL-6, IL-1*β*, TNF-*α*, IL-17, and *β*-chemokines (CCL2, CCL3, CCL4, CCL7) across species at the transcriptional and protein levels indicates activation of converging inflammatory pathways, including NLRP3 inflammasome signaling, NF-*κ*B activation, and MAPK pathway dysregulation. Additionally, marked increases were observed in CXC chemokines (CXCL1, CXCL2, CXCL5, CXCL10) and colony-stimulating factor 2 and 3, which displayed the highest fold changes. Interestingly, CXCL5 has been shown to orchestrate the recruitment and spatial organization of innate and adaptive leukocytes in the lung during influenza infection, highlighting its role in regulating local inflammatory responses^188^.

Notably, this cytokine and chemokine signature is reproducible across independent studies and experimental models^119^^,^^134^^,^ arguing against experimental artifacts and supporting the presence of an LNP-associated inflammatory program.

### 4.5 Complement activation

Korzun et al.^134^ observed in the raw data, that all models used C3 activation with a 1.5-log fold change. This suggests that Complement 3 activation was not primarily driven by TLR4. G-protein coupled receptors, such as the C3a receptor, are highly dependent on cell membrane integrity and cholesterol ratios^189^, ^190^, ^191^, ^192^, ^193^, ^194^.

Interestingly, the study by Luo et al.^137^ investigated interactions between different ionizable LNP lipids (MC3, Lung SORT, SM-102, and ALC-0315) and cell surface proteins to identify potential competitors for LNP attachment forming a proteinaceous corona. In this context, vitronectin and ficolin-1 were found to particularly facilitate attachment of LNPs to heart tissue cells. Interactions with vitronectin may promote cell proliferation and enhance tumor growth and metastatic potential^195^, while ficolin-1, through binding to transforming growth factor (TGF-*β*1), may modulate the lectin pathway and the complement component of innate immunity^196^. This suggests that the protein corona itself may already have direct effects on membrane organization.

Notably, analysis of the raw data reported by Korzun et al.^134^ revealed that PIK3C2G, which encodes a class II PI3K, was consistently downregulated across all datasets, providing additional evidence in support of our L-DMD hypothesis. The combination of TLR4-independent C3 activation and evidence that protein corona components directly engage membrane-associated receptors supports the L-DMD model, in which LNPs can initiate immune signaling through biophysical membrane reorganization prior to, or in parallel with, classical receptor-mediated pathways.

### 4.6 Downregulation of PPAR and AMPK signaling

Given that peroxisome proliferator-activated receptor gamma (PPAR*γ*) is a central regulator of lipid metabolism, mitochondrial function, and anti-inflammatory signaling^119^, and is modulated through AMPK-dependent phosphorylation^197^^,^^198^, its strong downregulation at the transcriptional level suggests disruption of homeostatic lipid and energy control. Such suppression, as reported under inflammatory or stress conditions^199^, ^200^, ^201^, may exacerbate NF-*κ*B activation (as shown by the data) and contribute to chronic inflammation and/or unpredictable outcomes. The expression pattern is indicative of MAPK pathway dysregulation affecting PPAR*γ* phosphorylation and signaling^200^^,^^201^. Notably, in macrophages, PPAR*γ* regulates inflammatory gene expression^200^. Ballav et al.^202^ highlight that partial PPAR*γ* dysregulation can broadly disrupt cellular metabolism and signaling, influencing disease fate decisions across multiple tissues.

If such PPAR suppression were sustained across tissues, as documented in Section 2.2 (liver, spleen, lymph nodes), this would be consistent with the systemic, multi-organ distribution pattern of the LNPs and the L-DMD framework. However, direct evidence of persistent PPAR dysregulation in human tissues following modRNA-LNP administration is lacking, and extrapolation to chronic disease outcomes requires longitudinal validation.

### 4.7 RAS (Rat sarcoma) and MAPKs

In summary, the insights of Knabl et al.^138^ and Korzun et al.^134^ support the assumption that the inflammatory properties of LNPs observed in mice by Ndeupen et al.^119^ and the findings of Luo et al.^137^ are also present following BNT162b2 transfection. Moreover, not all immune cells internalize LNPs equally, as uptake efficiency is influenced by factors such as the protein corona and the modRNA payload (Section 2.2).

Such induction is consistent with stress-responsive transcriptional reprogramming. In this context, KRAS activation represents a central signaling hub, as RAS-driven pathways can engage, for example, both RAF/MEK/ERK, p38-MAPK pathways and PI3K/AKT cascades (which was also observed in the proteomics by Hickey et al.^139^), thereby integrating inflammatory, metabolic, and genotoxic stress signals and promoting short-term cellular adaptation. The concurrent upregulation of p53, as also reported by Knabl et al.^138^, suggests the activation of compensatory checkpoint mechanisms that counterbalance proliferative or stress-related cues^203^^,^^204^.

Growing evidence indicates that GTPases, particularly RAS isoforms and RHOA, are spatially localized at the plasma membrane, where they play crucial roles in vesicle trafficking and membrane organization. Additionally, these GTPases can interact with lipids of the inner membrane leaflet, including phosphatidylserine and PIPs, forming lipid-protein interfaces that may influence local signaling nanodomains^34^^,^^205^, ^206^, ^207^, ^208^, ^209^, ^210^.

The partial heterogeneity of transcriptional changes among different age groups highlights limitations in generalizing the transcriptomic effects observed in Knabl et al.^138^ and sets the stage for the subsequent discussion of more controlled and consistent experimental models.

However, these observations position RAS^211^^,^^212^ and other related GTPases, like RHO^213^^,^ as membrane–proximal integrators of phosphoinositide- and voltage-dependent signaling perturbations, providing a direct mechanistic bridge between membrane dysregulation and downstream transcriptional reprogramming, including the translation of altered electrical signaling into sustained cellular state changes^211^^,^^212^. Interestingly, Guillot-Ferriols et al.^214^ and Chen et al.^215^ demonstrated that electric fields affect intracellular signaling pathways.

### 4.8 Transcription factors (E2F1, E2F8) and mechanistic target of Rapamycin complex (mTORC)

Knabl et al.^138^ note: “*Interestingly E2F8, but not CCNA1 or E2F1, was modestly upregulated 7 days post-vaccination in the naïve vaccinated individuals (1.5-fold, padj = 0.04).*” However, growing evidence suggests that *E2F1/2/4* expression correlates with immune cell infiltration and may modulate immune responses in proliferative or stressed cells^216^, ^217^, ^218^. It is known that E2F1 is in tight crosstalk with the 7 other members of the E2F transcription factor family^219^, ^220^, ^221^ during cell cycle phases. Furthermore, it has been shown that E2F1 plays a crucial role in immune cell differentiation and cloning^222^^,^^223^.

These findings support the notion that modRNA-LNP exposure can induce selective, systemic transcriptomic changes even in the absence of active viral infection, with partial activation of cell cycle genes, interferon responses, and partial mTORC1 engagement without coherent downstream cell-cycle coordination, consistent with a fragmented or dysregulated cell cycle program rather than a synchronized proliferative response^224^^,^^225^.

Furthermore, the consistent active p53 upregulation in both age groups, together with a decoupled *E2F1* from *E2F8* transcription factor transcriptome, indicates that cell cycle dysfunctionality was observed in the immune cells, which depended on the mTORC1 signaling regulating *CDC25A*, *RRM2* and *BUB1*^226^^,^^227^. Another indication that the cell cycle is affected at the transcriptomic level is the coordinated enrichment of gene sets associated with mitosis, checkpoint control, and Rho-GTPase signaling. All analyses were performed using KEGG and showed that the signals were active.

Emerging evidence suggests that PI(3,5)P_2_ may act as an upstream lipid regulator by forming a triad of lipid–lipid, lipid–protein, and protein–protein interactions, thereby modulating PI3K class I signaling while being generated by PI3K class III and phosphoinositide kinase FYVE-type zinc finger-containing activity. These pathways are functionally linked to mTORC1 and mTORC2 regulation^228^, ^229^, ^230^.

This decoupled activation pattern indicates a state of cellular stress adaptation rather than controlled expansion, with potential long-term consequences for immune cell homeostasis and functional changes.

### 4.9 Conceptual consolidation of sections 3 and 4

Of note, Luo et al.^137^ also observed with empty LNPs the activation of eucaryotic initiation factors (eIF) family. Driven by these data, we suggest that signaling transduction pathways such as mTORC and the MAPKs regulating eIFs may also be involved in endosomal escape processes^231^^,^^232^.

However, *in vitro*, *ex vivo*, and *in vivo* mouse data demonstrate that lipid-formulated constructs, such as liposomes and LNPs, alter cellular transcriptomics and proteomics by modulating signal transduction pathways, receptor activation, and phosphorylation cascades^119^^,^^132^^,^^233^, ^234^, ^235^. This finding is of considerable significance, as the omics data we have discussed here, along with shifts in signaling pathways, cannot be attributed solely to the presence of modRNA and/or spike proteins.

This is reasonable, given the sequence of events: the LNPs are first taken up *via* membrane physics and chemistry (time point 0), after which modRNA is released into the cytosol with a delay. In fact, release rates, estimated to be at 10%–15% or less, as suggested by Müller et al.^42^, appear far more likely to indicate that most of the modRNA remains within the LNP and is subsequently exocytosed. Importantly, this delayed release and translation may occur at anatomical sites distant from the injection site (Section 2.3).

Our L-DMD hypothesis implies that LNPs may also drive the immune system's initial response through membrane-driven effects on transfected cells, thereby affecting systems beyond immune cells. This is further supported by the study by Connors et al.^236^, which showed that eLNPs induce activation and maturation of monocyte-derived dendritic cells (MDDCs) and upregulate CD40 expression, leading to the recruitment of pro-T follicular helper cell cytokines, IL-6, IL-12, and IL-21. These findings are also consistent with Amor et al.^237^. Furthermore, Qin et al.^233^ showed in mice that these effects have transgenerational immunological consequences.

Taken together, these findings indicate that LNP-driven immune activation is not restricted to acute innate immune responses or transient antigen-presentation-linked effects, but can also engage epigenetically mediated regulatory programs, consistent with recent evidence demonstrating short-term and persistent epigenetic memory in innate immune cells following BNT162b2 mRNA vaccination^238^^,^^239^. In this context, recent work by Chytła et al.^240^, for example, has highlighted that PI(4,5)P_2_ signaling is not confined to the plasma membrane but also operates at the nuclear level, where it contributes to transcriptional regulation and chromatin organisation^241^.

We next summarize the given evidence supporting our working hypothesis. Table 2^119^^,^^132^^,^^134^^,^^137^, ^138^, ^139^^,^^159^^,^^171^^,^^242^ organizes the omics-derived pathway alterations (Sections 3 and 4) into functional categories that reflect the proposed L-DMD mechanistic cascade. Primary evidence comes from the previously discussed omics studies, supported by human observations, to show the functional relevance of molecular findings in human populations. Data serves as supporting evidence of pathway engagement rather than as validated clinical evidence outcomes.

**Table 2:** Membrane-associated signaling pathways perturbed by LNP exposure.

Section	Pathway	Membrane connection	Representative evidence	Ref.
Membrane structure and maintenance				
4.1	ESCRT/Endocytosis	• Membrane repair following endosomal stress. • Recruitment depends on phosphoinositides.	H-Px, ↓ESCRT proteins and ↓endocytic activity after modRNA-LNP injection.	Hickey et al.^139^
4.2	CYP	• ER/mitochondrial embedded membranes. • Catalytic geometry depends on local phospholipids and charge.	↕ M-Tx (eLNP), H-Px, H-Obs, ↓CYP metabolism post i.m. injection in mice; ↑Phase I enzymes (♂ only, BNT162b2 specific, hepatocellular leakage^a^), clozapine toxicity post-vaccination (functional corroboration^b^).	Ndeupen et al.^119^ Hickey et al.^139^ Thompson et al.^159^
Innate immune signaling				
4.3	TLR4 axis (MyD88/TRIF bias)	• Plasma lipid membrane organization. • Intact phosphoinositides needed for balanced signaling.	M-Tx (eLNP), M-Fx, MyD88-dominant NF-*κ*B activation, reversible with TLR-4 blockade (TAK-242) in human THP-1 cells *in vitro via* NF-*κ*B.	Ndeupen et al.^119^ Korzun et al.^134^ Zelkoski^c^ et al.^132^
4.4	Pro-inflammatory cytokines	• Membrane-associated PRR signaling (NF-*κ*B/MAPK). • Functional organization of opes within inner-leaflet lipid raft.	↕ (M-Tx, M-Tx (eLNP)), H-Obs, ↑NF-*κ*B targets and canonical cytokines in murine eLNP, Concordant upregulation in human datasets, cytokine-mediated drug interactions documented.^d^	Ndeupen et al.^119^ Korzun et al.^134^ McColl et al.^242^
4.4	Β-Cytokines, CXC- chemokines, IL-6, IFNs (across species)	• Membrane proximal NF-*κ*B activation.	↕ (MTx, MFx (eLNP)), H-Tx, Concordant CCL family upregulation in murine eLNP and human buffy-coat transcriptomes (CXCL 9,10).	Ndeupen et al.^119^ Korzun et al.^134^ Knabl et al.^138^
4.5	Complement (C3, lectin)	• Membrane-bound GPCR receptors (C3aR). • Protein corona effects.	M-Tx (eLNP), ↑C3. M-Px, LNP-vitronectin/ficolin-1 binding facilitates C3 activation events.	Korzun et al.^134^ Luo et al.^137^
Growth and metabolic signaling				
4.7	RAS–RAF–MEK–ERK (MAPK)	• RAS GTPases anchor lipids at plasma membrane nanodomains. • Translate local charge shift to signal amplification.	↕ M-Tx (eLNP), H-Tx, H-Px, MAPK pathway upregulation across species omes.	Korzun et al.^134^ Knabl et al.^138^ Hickey et al.^139^
4.7	PI3K/AKT/mTOR	• Membrane-localized PI3K. • Partial mTORC1 engagement indicates upstream dysregulated lipid signaling *via* RAS.	↕ M-Tx (eLNP), H-Tx, H-Px, Partial MAPK pathway + PI3K modulation post LNP exposure.	Korzun et al.^134^ Knabl et al.^138^ Hickey et al.^139^
4.4	PI3K class II	• Often activated downstream of RTKs + GPCRs. • Act independently of regulatory subunits characteristic of PI3Ks.	M-Tx (eLNP), Consistent ↓*Pik3c2g* across datasets.	Korzun et al.^134^
4.6	PPAR*γ*	• Integrates lipid-derived signals. • Suppression favors NF-*κ*B activation.	↕ M-Tx (eLNP), H-Obs, ↓PPARγ pathway → loss of anti-inflammatory tone + lipid metabolism imbalance, post-vaccination hypercholesterolemia (OR, 1.54, 95% CI: 1.36–1.74; population level corroboration)^e^.	Ndeupen et al.^119^ Huang et al.^171^
4.6	AMPK	• Senses membrane-linked stress. • Suppression favors NF-*κ*B activation.	M-Tx (eLNP), ↓AMPK → reduced oxidative metabolism and ↑NF-*κ*B.	Ndeupen et al.^119^
Cell cycle and stress response				
4.8	p53 + *E2F* Network	• Downstream of RAS–MAPK and PI3K–mTOR signaling. • Cell-cycle checkpoint.	H-Tx: p53 + *E2F8,* partial mTORC1 activation.	Knabl et al.^139^

Section numbers correspond to their respective sections in the main text.

H, human, M, mouse; Tx, transcriptomic; Px, proteomic; Obs, observational; Fx, functional; ↕, cross-species concordance (human + mouse); AKT, protein kinase B (also PKB); AMPK, AMP-activated protein kinase; C3, complement component 3; C3aR, complement C3a receptor; CAR, constitutive androstane receptor; CCL, C–C motif chemokine ligand; CI, confidence interval; CXCL, C–X–C motif chemokine ligand; CYP, cytochrome P450 enzyme; E2F, E2 transcription factor family; eLNP, empty lipid nanoparticle (no mRNA payload); ER, endoplasmic reticulum; ERK, extracellular signal-regulated kinase; ESCRT, endosomal sorting complex required for transport; GPCR, G protein-coupled receptor; GTPase, guanosine triphosphatase; i.m., intramuscular administration; KRAS, Kirsten rat sarcoma viral oncogene homolog; MAPK, mitogen-activated protein kinase; MEK, mitogen-activated protein kinase kinase (also MAPK/ERK kinase); modRNA–LNP, nucleoside-modified mRNA formulation encapsulated in a lipid nanoparticle; mTOR, mechanistic target of rapamycin; mTORC1, mechanistic target of rapamycin complex 1; MyD88, myeloid differentiation primary response 88; NF-*κ*B, nuclear factor kappa B; OR, odds ratio; p53, tumor protein p53; PI3K, phosphatidylinositol 3-kinase; PIK3C2G, phosphatidylinositol-4-phosphate 3-kinase catalytic subunit type 2 gamma; PIP, phosphatidylinositol phosphate; PM, plasma membrane; PPAR*γ*, peroxisome proliferator-activated receptor gamma; PRR, pattern recognition receptor; PXR, pregnane X receptor; RAF, rapidly accelerated fibrosarcoma (serine/threonine kinase); RAS, rat sarcoma; RTK, receptor tyrosine kinase; TLR4, toll-like receptor 4, TRIF, toll/interleukin-1 receptor/resistance protein (TIR)-domain-containing adapter-inducing interferon-*β*.

a Sex- and vaccine-specific enzyme elevation without CAR/PXR indicates leakage, rather than de novo synthesis^139^.

b Clinical corroboration: Clozapine (CYP1A2/3A4 substrate) toxicity post vaccination supports enzyme suppression observed in omics datasets. Observational data included as supportive evidence of pathway engagement.

c Not Omics data but included as supportive evidence for the mechanistic inference.

d Cytokine-mediated mechanism reviewed by McColl et al.^242^; observational reports provide complementary validation.

e Population-based association for lipid metabolic imbalance; causality requires further investigation.

Table 3^119^^,^^134^^,^^137^, ^138^, ^139^ presents primary omics and functional studies that inform the pathway classifications in Table 2. Each record details the model, material type, route of exposure, data modality, and timeline used to resolve membrane-associated signaling alterations described within the L-DMD framework.

**Table 3:** Summary of omic studies informing Table 2 (model, methods, route, and data type).

Study and reference	Model/species	Material tested	Route/dose	Data type/readout	Timeline/endpoints
Ndeupen et al.^119^	Mouse (C57BL/6, WT).	eLNP (no mRNA).	i.m. 10 μg in PBS.	M-Tx, (Whole tissue RNAseq + GSEA) -seq + GSEA.	∼24 h post injection.
Korzun et al.^134^	Mouse (WT + KO lines).	Luc mRNA LNP + eLNP.	Luc mRNA + eLNP groups received 5 μg in 100 μl PBS *via* i.p. as a single dose (acute) or i.p. once every 24 h for 3 doses (chronic). *n* = 4 groups total. eLNP groups received TAK-242 inhibitor 2 h prior to each dose.	M-Tx, M-FX, (behavior + ELISA + Transcriptome).	6 h post last dose.
Luo et al.^137^	Mouse/cell models.	eLNP (no payload) + comparators.	Multiple routes (i.n., i.m., i.v., PO, ID), 0.0005–0.5 mg/kg.	M-Px, (quantitative proteomics).	≤24 h post dose.
Hickey et al.^139^	Human (adults post BNT162b2/mRNA1273 vaccination)^a^.	modRNA-LNP vaccination.	i.m., clinical dose.	H-Px, (SomaScan v4.1 proteomics).	1 month and 6 months post-dose dose 3.
Knabl et al.^138^	Human (patients and healthy volunteers)^a^ Elderly patients (3 doses, postvaccination or infection, treatment with dexamethasone) Younger healthy group (naive, 1st dose).	modRNA-LNP vaccination/infection cohort.	i.m., clinical dose.	H-Tx, (whole blood buffy coat transcriptomics)	Elderly, Days 7–60. Younger, Days 7–10.

C57BL/6, inbred mouse strain C57 black/6; eLNP, empty lipid nanoparticle (no mRNA payload); Fx, functional assays; GSEA, gene set enrichment analysis; H, human; i.m., intramuscular administration; i.p., intraperitoneal administration; i.v., intravenous administration; KO, knockout; Luc, luciferase; SQ, subcutaneous administration; i.n., intranasal administration; M, mouse; PO, oral administration; ID, intradermal administration; Px, proteomic profiling; seq, sequencing (or sequence data); Tx, transcriptomic profiling; WT, wild type.

a Post-vaccination samples; Knabl cohort included patients with concurrent SARS-CoV-2 Beta-variant infection, potentially confounding attribution to LNP-only effects. Hickey cohort represents healthy adults post 3rd dose.

Taken together (Tables 2 and 3), these findings suggest that the earliest perturbations following LNP exposure likely originate at the plasma membrane, with the PI cycle as a central regulatory hub. Even subtle disturbances can cascade through lipid raft organization, receptor localization, and downstream signaling, producing transcriptomic and proteomic alterations, as shown by Hickey et al.^139^ and Luo et al.^137^.

Establishing precise *in vivo* mechanistic patterns remains challenging, especially without large cohorts stratified by age, vaccine type, dose, batch, comorbidities, prior illnesses, genetic predisposition, and LNP formulations. Extrapolation to chronic outcomes (*e.g.*, autoimmunity^243^, myocarditis^117^) requires longitudinal validation. The pathway enrichments presented here are hypothesis-generating rather than definitive disease mechanisms.

Omics data from Sections 3 and 4 demonstrate multi-pathway perturbations following LNP exposure that cannot be attributed to the payload alone. Convergent alterations–including phosphoinositide signaling^139^, *Pi3kc2g* downregulation^134^, mTOR and MAPK engagement, ESCRT dysregulation^139^^,^^149^, and metabolic pathway suppression^119^ across studies and platforms support the L-DMD hypothesis.

These observations indicate that early plasma membrane perturbations propagate through lipid rafts, receptor localization, and downstream signaling cascades, resulting in the observed transcriptomic and proteomic changes. Such early membrane-centered dysregulation, though not reflected in conventional lab values, may initiate broader signaling alterations, including paracrine and endocrine effects^244^.

In the next section, we examine membrane organization, focusing on lipid rafts, endosomal-lysosomal, and autophagosomal aspects that may drive changes in gene expression in the PI3K/AKT/mTORC1^245^^,^^246^, JAK–STAT^247^^,^^248^, and MAPK^249^ cascades observed in the omics data.

## 5 Breaching the plasma membrane: important roles for phosphoinositides (PIPs)

In Section 2, we outlined that LNP/modRNA complexes enter cells *via* receptor-dependent and receptor-independent endocytosis-like mechanisms, including membrane breaching. LNPs transfect diverse cell types, including epithelial cells, B cells, T cells, macrophages, and dendritic cells, depending on formulation and *in vivo* transformations, though the contribution of individual cell types to COVID-19 modRNA-LNP transfection remains under investigation. Computational simulations show that various ionizable lipids can integrate into the inner plasma membrane leaflet^17^, ^18^, ^19^, making early membrane-level alterations of receptor localization or conformation mechanistically plausible. LNP uptake occurs *via* endocytosis and/or macropinocytosis, as demonstrated by Panariti et al.^250^, Voigt et al.^251^, and reviews by Wang et al.^252^ and Gerelli^253^.

Building on the phosphoinositide-centered signaling framework, we expand the hypothesis to include membrane biophysical and biochemical constraints that may couple LNP exposure to intracellular trafficking dynamics, integrating previously discussed multi-omics observations with membrane-scale processes. Membrane penetration and direct interactions of ionizable lipids with the bilayer and membrane-bound receptors may drive membrane reorganization events, including alterations in lipid raft stability, which serve as organizational platforms for receptors such as G protein-coupled receptors (GPCRs) and receptor tyrosine kinases.

This aligns with observations by Wang et al.^252^ and Aliakbarinodehi et al.^74^. Lavagna et al.^254^ suggest that size-dependent aggregation of hydrophobic nanoparticles within lipid membranes induces local curvature, folding, and transient phase transitions, acting as an initial mechanical trigger perturbing phosphoinositide organization and receptor localization, and setting the stage for altered signaling and recycling dynamics.

### 5.1 Brief overview of the phosphatidylinositol (PI)-cycle

**Figure 3:**
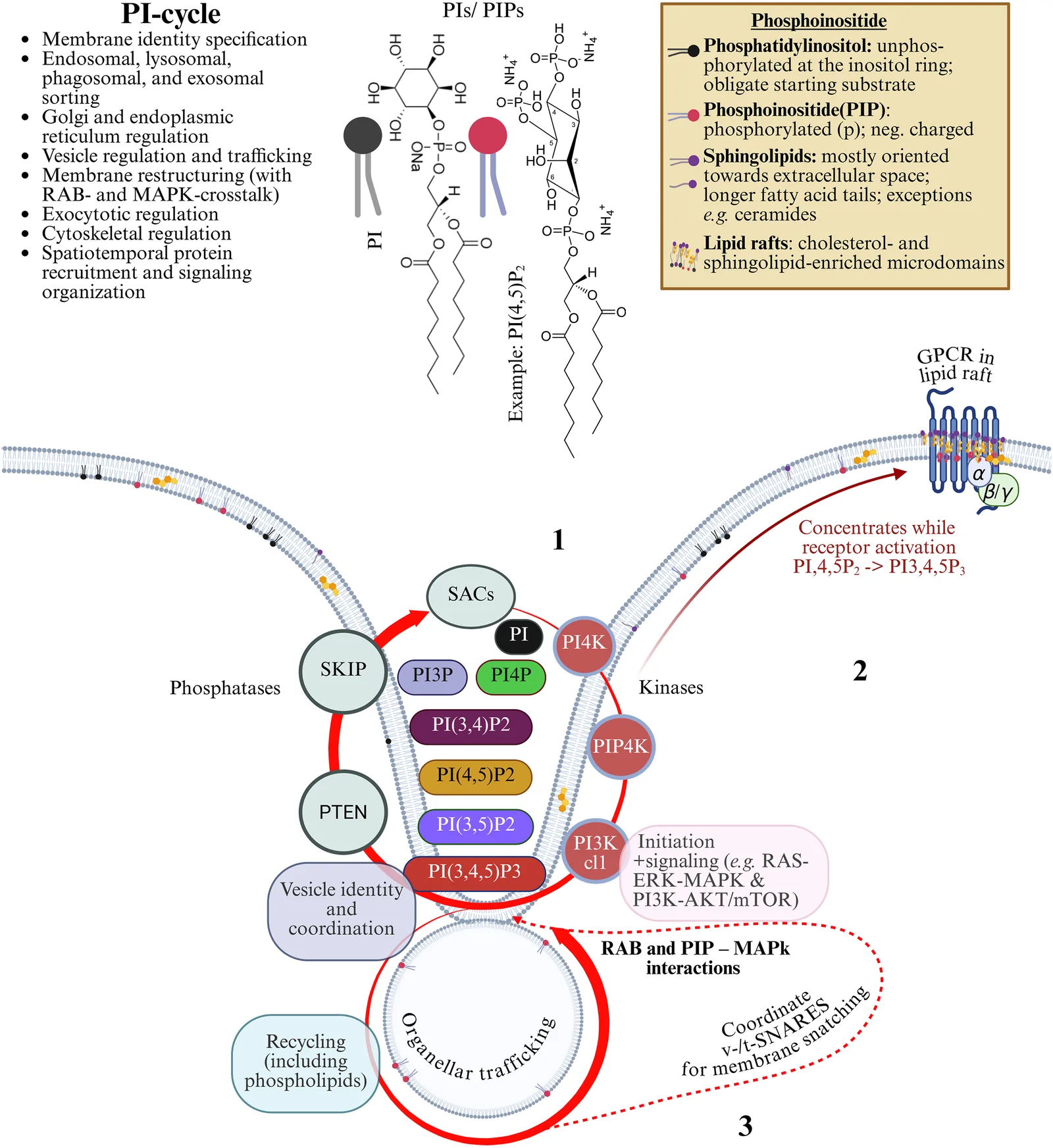
Cell membrane organization and the phosphatidylinositol (PI)-cycle. This schematic summarizes established PI-cycle biology and its role in defining membrane identity, vesicle trafficking, and endosomal maturation through spatially restricted phosphoinositide-species. Distinct phosphorylated PI pools coordinate receptor-proximal signaling and trafficking decisions, thereby linking membrane dynamics to downstream pathways such as PI3K–AKT–mTOR and RAS–ERK/MAPK signaling. (1) Phosphoinositide phosphatases remove phosphate groups, ensuring spatial and temporal signal termination within the PI cycle. (2) Phosphoinositide kinases generate specific phosphoinositide-species through site-specific phosphorylation of phosphatidylinositol. (3) Phosphoinositides cooperate with SNARE-proteins and small GTPases (*e*.*g*., RAB) to regulate membrane curvature, fusion, and biophysical properties that underlie vesicular transport and signal compartmentalization. Abbreviations: AKT, protein kinase B; ERK, extracellular signal-regulated kinase, GPCR, G-protein coupled receptor; MAPK, mitogen-activated protein kinase; mTOR, mechanistic target of rapamycin; mTORC, mechanistic target of rapamycin complex; PI, phosphatidylinositol; PIP, Phosphoinositide; PI4P, phosphatidylinositol 4-phosphate; PI(3,4)P_2_, phosphatidylinositol 3,4-bisphosphate; PI(3,5)P_2_, phosphatidylinositol 3,5-bisphosphate; PI(4,5)P_2_, phosphatidylinositol 4,5-bisphosphate; PI(3,4,5)P_3_, phosphatidylinositol 3,4,5-triphosphate; PI3K, phosphoinositide 3-kinase; PI3Kcl1, class 1 phosphoinositide 3-kinase; PI4K, phosphatidylinositol 4-kinase; PTEN, phosphatase and tensin homolog; SAC, sac domain-containing phosphoinositide phosphatase; RAB, ras-related in brain (GTPase family); RAS, rat sarcoma (GTPase family); SKIP, sphingosine kinase-interacting protein; SNARE, soluble NSF attachment protein receptors. Created in BioRender by Seger, F. (2026). https://BioRender.com/unux4hx.

Given that membrane interactions are evident, the membrane structuring and recycling process must be examined by investigating the PI-cycle (Fig. 3 and Supporting Information Table S1) and the potential effects LNPs may have on it.

The eukaryotic plasma membrane is a phospholipid bilayer forming a stable barrier between aqueous compartments^255^. Phospholipids are amphipathic, with hydrophobic tails oriented inward and hydrophilic heads facing the aqueous environment, thereby enabling selective transport. Membrane fluidity depends on fatty acid composition and cholesterol content. Embedded proteins, such as receptors (*e.g.*, GPCRs), ion channels, and transmembrane enzymes (*e.g.*, ACE2), mediate transport, signaling, and cell–cell recognition *via* extracellular signals^256^. Fatty acid composition often follows the 18:0/18:1 or 18:1/18:1 rule, where unsaturated chains (*e.g.*, 18:1) reduce packing density and increase lipid mobility^257^. The bilayer should be regarded as a fluid mosaic^258^.

Lipid rafts are specialized microdomains enriched in sphingolipids and cholesterol, with specific phospholipid composition, including phosphoinositides, influencing membrane order and permeability^259^^,^^260^. They create local spatial and electrical potential differences, promoting receptor clustering and anchoring of signaling molecules. Phosphoinositides act as first intracellular messengers, initiating downstream cascades at plasma and endosomal membranes after receptor internalization^261^. These cascades regulate the cycle *via* feedback loops [*e.g.*, *via* RHO-GTPases (Ras homologous) and cell division cycle 42 (Cdc42—part of the RHO family)] ^262^^,^^263^.

Fallahi-Sichani & Linderman^264^ showed that lipid rafts as membrane microdomains influence the localization of receptors like GPCRs. With rapid dimerization and monomerization, frequent partner changes lead to the formation of oligomeric receptor clusters. In addition, the effect of ligands varies broadly: “*while some GPCRs are stimulated to dimerize by ligands, others are inhibited-indicating a differential, ligand-dependent receptor organization.*“^264^.

It has been suggested that these membrane microdomains also play a critical role in mediating interactions with exogenous particles. They can facilitate interactions between LNPs and the cellular membrane, thereby enhancing intracellular uptake through endocytosis^265^. Furthermore, the specific organization of these domains improves recognition and internalization processes, coordinates receptor localization and membrane trafficking and influences downstream signaling events, thus highlighting their central role in understanding how LNPs influence cellular homeostasis.

Although not yet demonstrated *in vivo*, it is conceivable that lipid nanoparticles may interfere with autophagic lysosome reformation (ALR)^266^^,^^267^, a process essential for sustaining lysosomal homeostasis and autophagic flux^268^^,^^269^. Since ALR depends on phosphoinositide transitions^270^ and clathrin-mediated membrane remodeling^271^, the insertion of ionizable lipids into these organelles could compromise the delicate balance of lysosomal recycling. Such perturbations may not only disturb autophagic flux but could also contribute to organ-specific toxicities reported in the nanomedicine safety literature^108^.

### 5.2 Signaling through phosphorylation states of phosphatidylinositol

Phosphatidylinositols (PIs) are amphiphilic glycerophospholipids that insert into the plasma membrane with their inositol headgroups facing the cytosol. At physiological pH, PI is monoanionic (net charge∼ −1), constitutes ∼5%–10% of membrane phospholipids^272^^,^^273^, and serves as a structural membrane component. Phosphorylated derivatives, phosphoinositides (PIPs), are low-abundance signaling lipids (∼1%–5%) that remain membrane-bound, define organelle identity, and regulate trafficking, including endocytosis, phagocytosis, and receptor-mediated internalization.

The PI cycle, described by Posor et al.^274^ (Table S1 for detailed PIPs-transitions with enzymes and kinases involved), is a dynamic network of phosphorylation transitions, *e.g.*, PI(4,5)P_2_ ↔ PI(3,4,5)P_3_, controlled by kinases (*e.g.*, PI3K), phosphatases (*e.g.*, phosphatase and tensin homolog, inositol polyphosphate phosphatases), and downstream effectors like AKT. These transitions govern organelle maturation, endosomal–lysosomal trafficking, phospholipid recycling, and membrane integrity^274^, ^275^, ^276^, ^277^, integrating with Ras-related in brain (Rab) GTPases and signaling cascades such as AKT/mTOR and ERK. Exposure to LNPs is hypothesized to perturb membrane dielectrics, electrostatics and biophysics, phosphoinositide organization, and endocytic trafficking, with secondary effects on PI-dependent signaling and organelle identity^278^^,^^279^.

### 5.3 The LNP components and their effects

#### 5.3.1 Ionizable lipids

**Figure 4:**
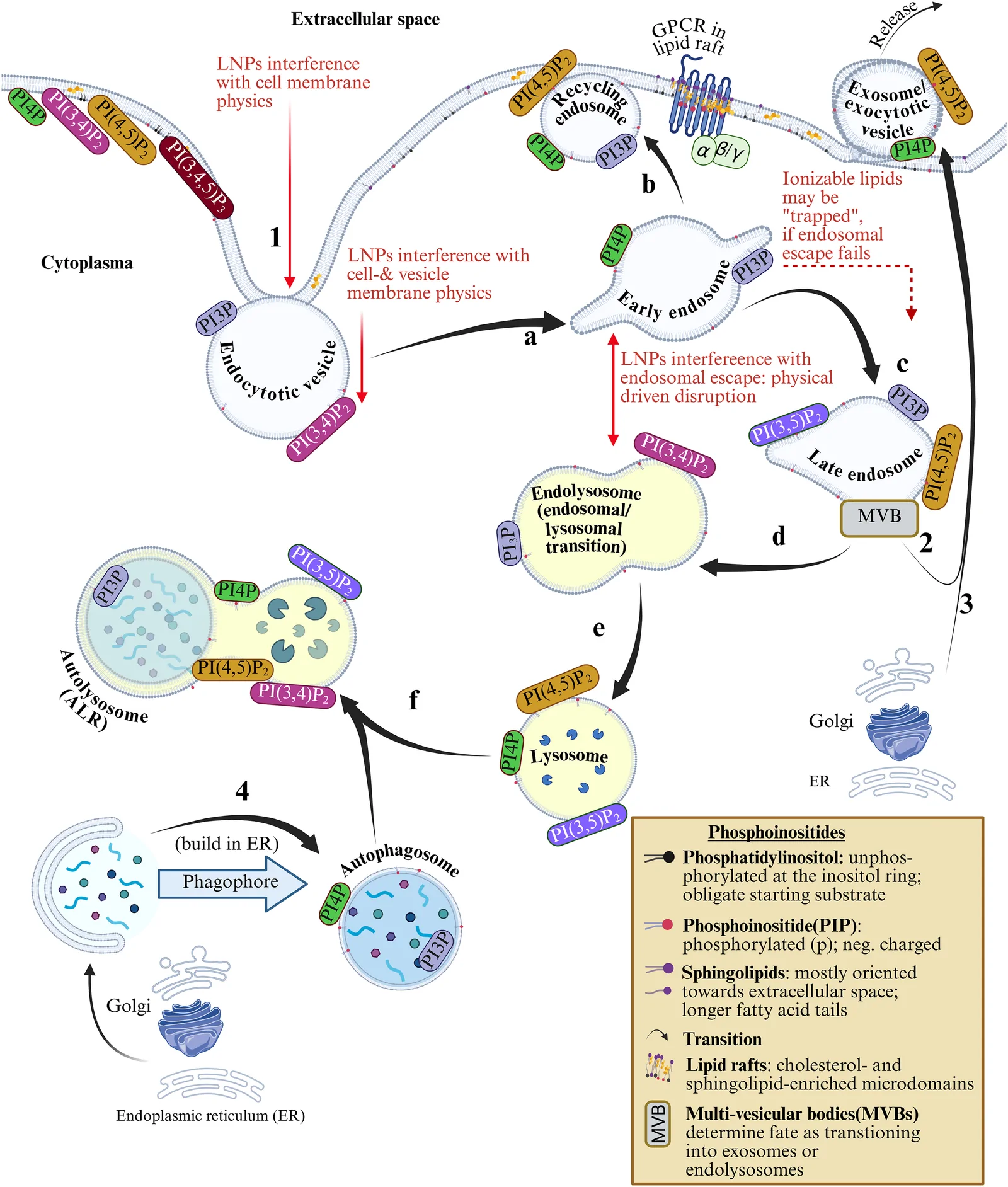
Organelle trafficking, organelle membrane identity, and lipid nanoparticle influence. (1) Internalization. Lipid nanoparticles are taken up by endocytosis and enter early endocytic vesicles. LNPs may interfere with physics of plasma and vesicle membranes during uptake and early endosomal trafficking. (a) Early endosome. Defined predominantly by PI3P, with contributions from PI4P. PI3P is generated by class III PI3K, which establishes endosomal identity by recruiting effector proteins, such as ESCRT-0 and SNX, for cargo sorting. (b) Recycling endosome. Enriched in PI(4,5)P_2_, PI4P, and PI3P, supporting membrane recycling, cargo resorting, and rebalancing of phosphatidylinositol species. (c) Late endosome. Characterized by PI3P with increasing levels of PI(3,5)P_2_, generated by PIKfyve. Regulates cargo sorting and MVB formation, and trafficking toward exosome release or lysosomal fusion. Incomplete endosomal escape may trap ionizable lipids within these compartments. (d) Endolysosomal transition. Characterized by elevated PI(3,5)P_2_ with residual PI3P supporting lysosomal maturation processes, including ion channel activity and V-ATPase function. (e) Lysosome. Dominated by PI(3,5)P_2_ and variable phosphoinositide species depending on functional state. Coordinates degradation of intracellular components and autophagic lysosomal reformation (ALR). (f) Autolysosome. Contains PI3P derived from autophagosomes and PI(3,5)P_2_ derived from lysosomes. During ALR, PI(4,5)P_2_ and PI4P are involved, with PI(4,5)P_2_ recruiting clathrin and the AP-2 complex to support membrane tubulation and lysosome reformation. (2) and (3) Exocytosis pathways. Cargo release occurs *via* MVB fusion with the plasma membrane or *via* the trans-Golgi network, involving PI4P and PI(4,5)P_2_, which regulate vesicle trafficking and exocytosis. (4) Autophagosome formation. Generated at the ER, PI3P produced by class III PI3K recruits WIPI2, promotes phagophore expansion and LC3 lipidation. Abbreviations: ALR, autophagic lysosomal reformation; AP-2, adaptor protein complex-2; ER, endoplasmic reticulum; ESCRT, endosomal sorting complexes required for transport; LC3, microtubule-associated protein 1 light chain 3; MVB, multivesicular body; PI3K, phosphoinositide 3-kinase; PI3K class III, class III phosphoinositide 3-kinase; PI3P, phosphatidylinositol-3-phosphate; PI3,4P_2_, phosphatidylinositol-3,4-bisphosphate; PI3,4,5P_3_, phosphatidylinositol-3,4,5-trisphosphate; PI3,5P_2_, phosphatidylinositol-3,5-bisphosphate; PI4P, phosphatidylinositol-4-phosphate; PI4,5P_2_, phosphatidylinositol-4,5-bisphosphate; PI(3,4,5)P_3_, phosphatidylinositol-3,4,5-triphosphate; PIKfyve, phosphoinositide kinase FYVE-type zinc finger-containing; SNX, sorting nexin; V-ATPase, vacuolar-type H^+^-ATPase; WIPI2, WD repeat domain phosphoinositide-interacting protein 2. Created with BioRender. Seger, F. (2026). https://BioRender.com/s8tc5vs.

Given that PIPs mark endosomal membranes and tightly regulate organelle maturation and trafficking, disruption of their dynamic phosphorylation-dephosphorylation cycles can compromise membrane integrity (Fig. 4). These lipids interact with negatively charged PIPs and the surrounding phospholipid matrix, potentially destabilizing the tightly regulated membrane environment described above. Consequently, this can induce transient endosomal and lysosomal membrane rupture or permeabilization, as demonstrated in recent studies, thereby facilitating the cytosolic release of encapsulated payloads, such as modRNA, while bypassing the canonical degradation pathways normally orchestrated by the PI cycle^88^^,^^149^. This is especially notable because only a proportion of payload-containing LNPs (depending on the formulation and cell type) reach the phase of endosomal/lysosomal escape^72^, and it is not yet known how many remain in endosomal/lysosomal/autophagosome transition^76^ (discussed in Section 2).

A recent spatiotemporal analysis^87^ using sensitive LNP labeling platforms further supports this notion, showing that continuous endocytosis and endolysosomal trafficking are required to maintain pools of releasable compartments, thereby mechanistically explaining why only a fraction of LNPs achieve cytosolic release while the remainder remain in lysosomes. This partial efficiency highlights the unresolved question of how the remaining LNPs, which do not induce endosomal rupture and are trafficked to lysosomes, are subsequently degraded or recycled within the cell, and whether their presence might disrupt these processes.

LNPs, upon endocytic uptake, introduce high local concentrations of cationic or ionizable lipids into the endosomal membrane, a phenomenon highlighted as a major determinant of both efficacy and toxicity of LNP formulations. Several studies, including Čechová et al.^17^, Ermilova and Swanson^18^, and Atmuri et al.^19^, have proposed that such lipids can trigger integrative membrane events. Jørgensen et al.^90^ further report that ionizable lipid–membrane interactions may lead to endosomal disruption, and that even biodegradation products of protonated tertiary amines-containing ionizable lipids, such as ALC-0315 and SM-102, remain bioactive. The authors note: “*However, they are neither synthesized from endogenous or natural structures nor are they degraded into typical biocompatible building blocks.*” How the integrative events^17^, ^18^, ^19^ relate to the direct disruption observed by Jørgensen et al.^90^ remains to be clarified.

#### 5.3.2 DSPC (1,2-distearoyl-sn-glycero-3-phosphocholine)

In the context of the recycling processes and membrane organization, another aspect that has received little attention is the phospholipid, DSPC^280^^,^^281^, which is used as a helper lipid in LNPs. DSPC (also known as 18:0/18:0) has two saturated stearic acid chains. While structurally similar to the phospholipids naturally found in plasma membranes, DSPCs are more tightly packed, more rigid, and more thermally stable^282^ than the typical 18:0/18:1 and 18:1/18:1 phospholipids found in eukaryotic membranes^257^^,^^283^. It has a high gel-liquid transition temperature (*T*_m_ approx. 55 °C), which gives it unusual rigidity^284^. It is a natural phospholipid, primarily found in human pulmonary surfactant^285^.

This raises the question of how DSPC affects the PI-cycle, given that phosphatidylcholines are recycled into various phospholipids, including PI. Phospholipase D, which converts phosphatidylcholines into phosphatidic acid, plays an essential role in this process^286^, ^287^, ^288^. Phosphatidic acid is converted into CDP-diacylglycerol by CDP-diacylglycerol synthase, a key step in channeling phosphatidic acid into the biosynthesis of PI and other phospholipids^273^^,^^289^. CDP-diacylglycerol subsequently serves as the immediate precursor for PI, synthesized by PI synthase at the endoplasmic reticulum, forming the core PI scaffold^273^^,^^290^.

Data on how DSPC is degraded and whether it can interfere with membrane physiology beyond the transfection process *via* recycling is sparse. After 24 h, most DSPC primarily accumulates in the liver, spleen, heart, kidney, and lung, indicating that these organs' plasma membranes take up the lipid^291^. This confirms DSPC's tissue delivery and retention. However, liposomes composed of phospholipids such as DSPC can markedly alter membrane structure, as previously discussed in the review by Lonez et al.^234^. Studies indicate that DSPC-containing liposomes can undergo hydrolytic and oxidative degradation, producing phospholipid-derived products that may alter membrane structure and stability in unpredictable ways^292^^,^^293^. However, the exact cellular mechanisms *in vivo* remain under investigation.

#### 5.3.3 Oxysterol-binding proteins

Oxysterol-binding protein-related proteins coordinate PI metabolism with sterol transport^294^^,^^295^, adding regulatory complexity to the PI cycle. Whether LNP-delivered cholesterol engages this pathway, particularly under inflammatory conditions^296^ that alter sterol trafficking and phosphoinositide turnover, warrants systematic investigation to determine if oxysterol-binding protein-related proteins mediate lipid exchange or signaling events.

#### 5.3.4 A role for lipid impurities

As we suggested in Section 2, LNPs act as bioactive particles with unpredictable biodistribution and transfection pathways. This makes it difficult to determine the membrane conditions under which their proposed mechanisms of action will occur. Furthermore, the process by which lipid impurities or their by-products may influence endosomal-lysosomal transition, autophagosome formation, and the subsequent autophagosome-lysosome fusion that leads to autolysosome formation requires further study.

Covalent RNA-lipid adducts formed during manufacturing^94^^,^^95^, and possible oxidative degradation products (*e.g.*, 4-HNE), may compound membrane perturbations (see Section 2.6). In particular, 4-HNE, a highly reactive *α*,*β*-unsaturated aldehyde generated from peroxidation of omega-6 polyunsaturated fatty acids, readily forms Michael adducts with nucleophilic amino acid residues (cysteine, histidine, lysine) in proteins^297^^,^^298^ If sufficiently generated *in vivo*, 4-HNE and related electrophilic lipid species merit systematic investigation as they are established modifiers of MAPK components and other signaling pathways^297^^,^^298^. Such modification could disrupt phosphoinositide homeostasis, whether by altering PI(4)P pools at the Golgi, impairing PI(4,5)P_2_-mediated membrane dynamics, or modulating PI(3,4,5)P_3_ signaling cascades that control vesicle trafficking, autophagy, and immune sensing, or directly modifying phosphoinositide-metabolizing enzymes that may contribute to sustained pathway dysregulation beyond direct membrane perturbations.

### 5.4 Small perturbations can lead to major shifts in PIP signaling

Recent work by Fung et al.^22^ demonstrates that phosphoinositide (PIP) interconversion is governed by feedback and feedforward circuits, excitable and oscillatory regimes, and kinetic thresholds, such that minor perturbations in membrane composition, enzyme activity, or lipid transport can trigger disproportionately large and sometimes paradoxical shifts in PIP balance. This nonlinearity implies that local LNP-associated perturbations (*e.g.*, ionizable lipids, DSPC, or pH changes) may push membranes across critical dynamical thresholds in downstream signaling^299^, altering endosomal identity, trafficking, and payload release. Consequently, simple formulation optimization may not ensure predictable delivery across diverse cell types or physiological states.

This perspective highlights that LNP-induced effects are not purely additive but may exceed critical system thresholds that conventional toxicological frameworks^299^, which often inadequately model complex, self-assembling bioactive nanosystems, may fail to capture. Models of lipid nanoparticle activity that neglect intracellular phosphoinositide processing and downstream signaling cascades, including MAPK pathways, therefore remain incomplete. Fung et al.‘s^22^ findings furthermore align with broader evidence that PIPs function as central hubs integrating signaling and mechanical regulation through feedforward and feedback interactions with cytoskeletal dynamics and membrane remodeling^300^, ^301^, ^302^.

## 6 Discussion

In this hypothesis-driven review, we proposed lipid nanoparticle-driven membrane dysfunction (L-DMD) as a mechanistic framework that may explain the persistent biological effects reported after exposure to LNPs. The model focuses on the bioactive properties of the ionizable lipid components and their interactions with cell membranes independent of any nucleic acid payload. In contrast to models which are limited to innate immune activation or modRNA translation kinetics, L-DMD emphasizes how direct physicochemical interactions with cellular membranes can induce sustained dysregulation in cellular signaling, metabolism, and homeostasis.

The core premise of the L-DMD hypothesis is that the metastable, supramolecular nature of LNPs, together with the electrostatic properties of ionizable lipids, can allow them to integrate into cellular membranes, including disruption of endosomal vesicles. This could lead to local changes in charge density, lipid packing, and the dielectric environment. Even minor alterations in these parameters may perturb localized pools of key phosphoinositides (*e.g.*, PI(4,5)P_2_, PI(3,4,5)P_3_, PI4P, PI(3,4)P_2_), which are central to membrane–proximal signaling and organelle identity. Therefore, their dysregulation can propagate to downstream pathways including NF-*κ*B, MAPK, JAK/STAT, and mTORC1/2, and through bidirectional feedback affecting upstream signaling nodes. This leads to the broad inflammatory stress responses and metabolic patterns observed in cellular, transcriptomic, and proteomic analyses.

Experimental lipidomic analyses of nanoparticle–plasma interactions support lipid-mediated membrane remodeling at the nano–bio interface. To the best of our knowledge, only Pepafilippou et al.^303^ have examined *ex vivo* lipidomic profiles of liposome-corona assemblies. Using HSPC/cholesterol liposomes incubated with human plasma, they identified selective enrichment of sphingomyelins, ceramides, and acylglycerols in the corona by mass spectrometry. Although not directly transferable to ionizable LNPs due to their pH-dependent biophysics, the study demonstrates selective lipid–lipid interactions at the nano-bio interface and supports the conceptual basis of our hypothesis.

**Figure 5:**
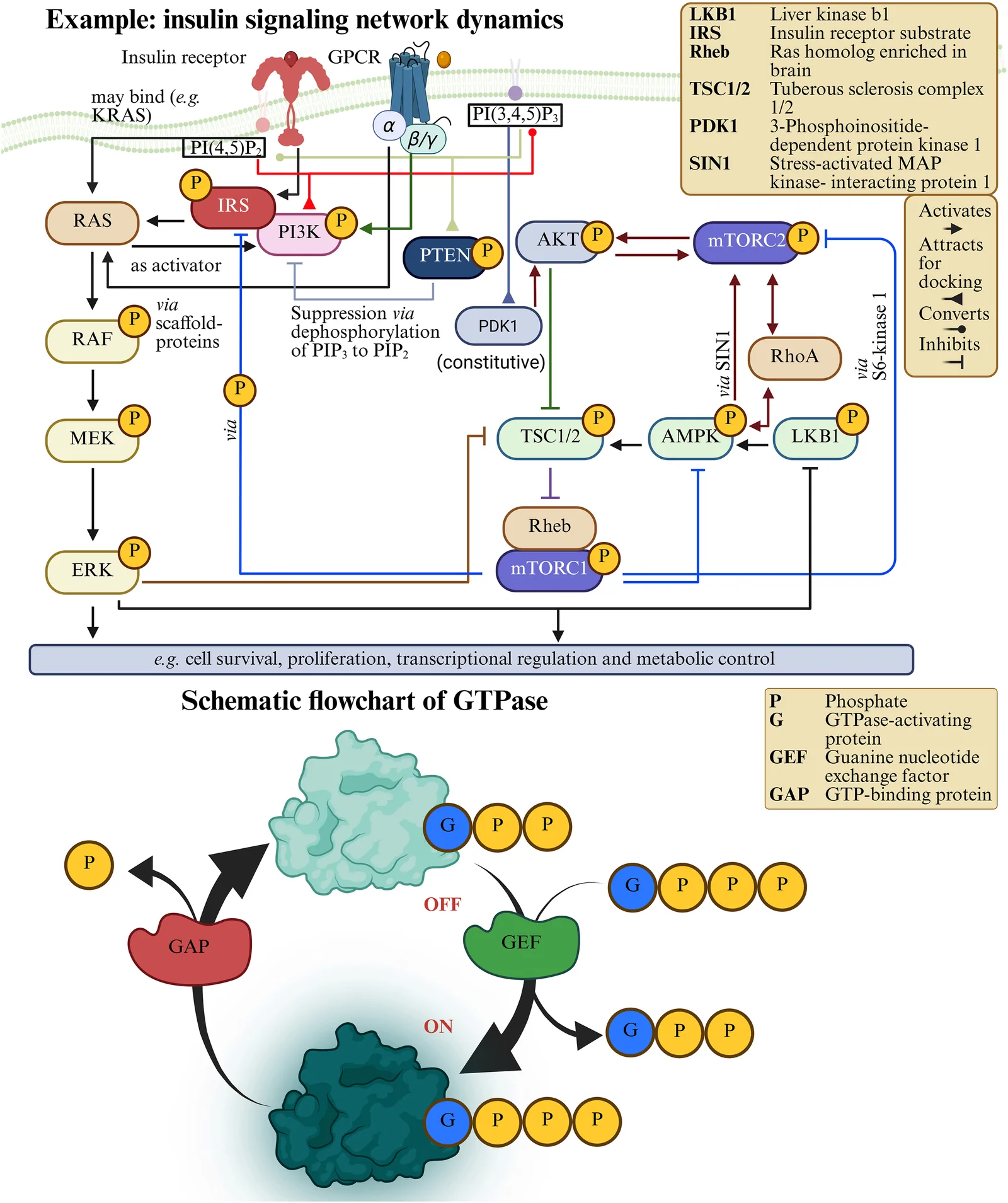
Membrane-organized phosphoinositide regulation: insulin pathway as an example. Dynamic routes of inhibition and activation are integrated through multi-layered feedback loops: mTORC1 acts as a central homeostatic regulator, providing inhibitory feedback to both the upstream IRS scaffold and the autocrine mTORC2–RHO–AMPK signaling axis to ensure signal termination and metabolic stability. The molecular logic of these transitions is governed by the GTPase cycle (GEF/GAP) illustrated below, which functions as the fundamental binary switch for network activation. GPCR activation at the plasma membrane, involving heterotrimeric G-protein subunits (G*α*, G*β*, and G*γ*), triggers PI3K-dependent conversion of PIP_2_ to PIP_3_, thereby linking receptor signaling to downstream AKT/mTOR and RAS/ERK cascades that govern cell growth, survival, and metabolic control. Insulin receptor activation triggers phosphorylation of the IRS scaffold, which recruits PI3K and Grb2/SOS to initiate the AKT and RAS/ERK cascades. This axis is linked to the GPCR system *via* RGS proteins that modulate G-protein activity, ensuring the synchronized control of metabolic and proliferative cellular responses. Note: This diagram is a simplified representation of the insulin signalling network, highlighting key regulatory axes rather than providing an exhaustive map of all participating molecules. Abbreviations: AKT, protein kinase B; AMPK, AMP-activated protein kinase; ERK, extracellular signal-regulated kinase; GBP, GTP-binding protein; GAP, GTPase activating protein; GEF, guanine nucleotide exchange factor; GPCR, G protein-coupled receptor; GRB protein 2, growth factor receptor-bound protein 2; IRS, insulin receptor substrate; LKB1, liver kinase B1; MEK, MAPK/ERK kinase; mTORC1/2, mechanistic target of rapamycin complex 1/2; P, phosphate; PDK1, 3-Phosphoinositide-dependent protein kinase 1; PI3K, phosphoinositide 3-kinase; PIP_2_, phosphatidylinositol-4,5-bisphosphate; PIP_3_, phosphatidylinositol-3,4,5-trisphosphate; RAF, rapidly accelerated fibrosarcoma kinase; RAS, rat sarcoma (small GTPase); RGS, regulators of G-protein signaling; Rheb, ras homolog enriched in brain; RhoA, Ras homolog family member A (small GTPase); SIN1, stress-activated map kinase-interacting protein 1; SOS, son of sevenless homolog; TSC1/2, tuberous sclerosis complex 1/2. Created in BioRender. Seger, F. (2026). https://BioRender.com/m6o2bva.

Signal transduction through PI3K/AKT/mTOR and RAS/ERK provides an example of this principle. These cascades are nonlinear, threshold-dependent systems with feedforward and feedback loops, where small shifts in lipid composition or charge distribution can destabilize network homeostasis^304^, ^305^, ^306^. One example, in which LNP-driven alterations at the plasma membrane or endosome could theoretically affect both the amplitude and duration of signaling outputs is shown schematically in Fig. 5 (*e.g*., insulin signaling pathways).

This framework addresses an existing explanatory gap in current pharmacodynamic and toxicological models, which often treat LNPs primarily as delivery vehicles and attribute observed effects primarily to the persistence of the modRNA-encoded protein^85^^,^^307^ or to transient innate immunity^308^^,^^309^.

The phenomenon of persistent detection of spike protein and modRNA in certain individuals is consistent with this framework and is of particular interest to us. L-DMD suggests that such persistence may arise from signaling dysregulation rather than intrinsic molecular stability. If LNP-induced perturbations create conditions under which MAPK signaling platforms activation becomes spatially and temporally dysregulated^19^, such dysregulation could, in principle, influence RNA turnover, translation shutdown and protein degradation mechanisms, producing functional persistence of modRNA and prolongation of protein half-life, even in the absence of chemical “indestructibility”^310^, ^311^, ^312^, ^313^, ^314^. We assume that the decisive factor is not the intrinsic chemical stability of modRNA, but a systems-level failure of cellular homeostatic shutdown mechanisms. As detailed in Section 3, the data point to alterations in phosphoinositide-regulated signaling nodes and do not provide evidence for a single dominant upstream trigger, leaving open the possibility of effects on higher-order signal termination processes.

Experimental human *ex vivo* data support this interpretation^315^. Sequence-optimized, but chemically unmodified PpLuc mRNA formulated in LNPs demonstrated significantly higher luciferase expression at 24 and 48 h compared with unencapsulated, uridine-unmodified mRNA, suggesting that the supramolecular colloidal structure of the mRNA–LNP complex enhances intracellular persistence.

Furthermore, activation of innate immune sensors such as Toll-like receptor 4 (TLR4) may be associated with modulation of intracellular RNA processing and proteostatic pathways. Experimental studies demonstrate that RNA sensing by endosomal Toll-like receptors requires coordinated RNase-dependent processing, including RNase T2, RNase 2, and OAS-RNase L systems, linking innate immune activation to RNA turnover regulation^316^, ^317^, ^318^. In parallel, TLR4 signaling has been shown to interact with proteolytic and ubiquitin-dependent protein regulatory networks^319^, ^320^, ^321^. Comparable innate immune activation has also been described for cationic lipid transfection systems such as Lipofectamine, supporting the concept that lipid-mediated immune sensing may influence intracellular RNA and protein processing dynamics^322^.

Together, these findings may indicate a shift in cellular homeostatic regulation rather than effects solely attributable to nucleoside modification. They support our argument that the functional behavior of modRNA LNP complexes can be influenced by supramolecular and biophysical properties, including entropic aspects of lipid organization, lipid–RNA interactions, phase behavior, and interactions with membrane-bound receptors and with plasma and vesicle membranes, which may in turn affect intracellular processing, RNA stability, and protein persistence.

The L-DMD hypothesis is testable with current tools. Endosomal function and LNP escape can be quantified using galectin-8-GFP or SNAPSwitch reporter assays and confocal microscopy; proteasome and RNase activities can be measured using Proteasome Glo kits, RNase L substrates, or pulse-chase experiments with actinomycin D, each under the control of MAPK inhibitors (*e.g*., SB203580, U0126) or mTORC blockers (rapamycin). KEGG pathway enrichment analyses of RNA-Sequence data (6–48 h post-LNP transfection) can reveal dysregulation of MAPK, mTORC, RNA degradation, and ribosome biogenesis pathways, complemented by phospho-Western blots for key proteins (p-ERK, p-p38, p-S6K).

In *in vivo* mouse models, bioluminescence imaging combined with tissue Quantitative Polymerase Chain Reaction/Enzyme-Linked Immunosorbent Assay enable the correlation of prolonged luciferase or spike persistence with individual signaling variability. By comparing modified *vs*. unmodified mRNA, empty LNPs, and genetic/pharmacological interventions, it is possible to distinguish effects driven by the supramolecular properties of the LNPs from those linked to the modRNA payload.

Variability in spike/modRNA persistence across individuals may reflect genetic or physiological differences in membrane signaling tolerance, feedback-loop robustness, or stress adaptation thresholds. These factors determine the rate at which a cell restores homeostasis after LNP-induced perturbations. Individuals with reduced ability or tolerance for membrane asymmetry or weaker feedback control could experience prolonged signaling activity and delayed molecular turnover.

Current post-marketing pharmacovigilance frameworks^323^ are optimized for detecting and attributing immunologically mediated adverse events with acute temporal profiles, typically within hours to weeks post-vaccination. The L-DMD hypothesis, however, predicts a distinct class of lipid-mediated responses characterized by low-grade activation of signaling pathways, phosphoinositide dysregulation, autophagy impairment, and metabolic imbalance, manifesting as delayed (weeks to months), cumulative, multi-systemic effects of mild-to-moderate severity. These attributes fall outside standard case definitions, temporal windows, and biological plausibility assessments linked to known immune mechanisms and are associated with high confounding in causality assessment in multifactorial pathology^323^^,^^324^. Empirical testing of L-DMD requires mechanism-informed biomarker development and prospective cohorts designed to detect non-immunological, delayed manifestations that current surveillance systems are not designed to capture.

Recognizing LNPs as active biointerfaces rather than inert delivery vehicles may enable the rational design of next-generation LNPs with improved safety profiles, enhanced controllability, and tunable persistence characteristics. Insights from the L-DMD hypothesis can guide three strategic directions.

1. Rational LNP design

Adjusting ionizable lipid composition, charge density, and membrane affinity could allow decoupling of delivery efficiency from prolonged signaling perturbation, enabling safer and more predictable nanomedicine platforms.

2. Spatiotemporal control of signaling

Determining how LNPs influence membrane signaling domains may facilitate deliberate tuning of MAPK, mTOR, or innate immune pathways for therapeutic benefit, including cancer immunotherapy or transient immune modulation.

3. Predictive biomarker development

Establishing readouts for phosphoinositide dynamics, MAPK deactivation kinetics, and ubiquitin–protease activity as candidate biomarkers to assess long-term cellular responses to LNP exposure.

L-DMD is important beyond modRNA vaccines. Similar lipid-based nanocarriers are increasingly used in gene therapy, oncology, and protein replacement medicine. Understanding how supramolecular lipid organization governs intracellular network behavior will likely have broad implications for nanomedicine, integrating biophysics, physicochemistry, pharmacology, and systems biology. Since open systems like LNPs operate *in vivo* alongside open systems like cells, this perspective may be useful for developing a consistent model^325^, ^326^, ^327^, ^328^ for future advancements.

Future research should prioritize analytical frameworks that characterize the supramolecular properties of LNPs, which are not fully addressed by current or emerging compendial standards^329^^,^^330^. While recent regulatory guidelines establish quality control parameters, including particle size, encapsulation efficiency, and chemical modifications such as adducts, they do not systematically assess biophysical determinants, such as ionizable lipid protonation state^9^, lipid domain organization^48^, membrane fusion kinetics, or structural heterogeneity^331^, that may directly influence the membrane perturbations of the L-DMD hypothesis. Advances in structural and functional assays, including cryo-EM, small-angle X-ray and neutron scattering, and single-particle fluorescence methods, provide nanoscale resolution of lipid packing and particle morphology, informing next-generation quality control strategies^48^^,^^332^.

*In vivo*, emerging tracking technologies, such as SPARKLE^61^, alongside mass spectrometry imaging, enable spatiotemporal resolution of LNP particle biodistribution that current fluorescent labeling cannot achieve without altering particle biophysics. Standardizing these methodologies will be essential for testing L-DMD predictions, identifying biomarkers of membrane dysregulation, and rationally engineering next-generation LNPs with improved safety and controllability. We see this as one of the largest gaps and one of the greatest opportunities for optimizing future research.

While the L-DMD framework raises important questions about long-term cellular responses to LNP exposure, it remains a hypothesis-generating model awaiting rigorous experimental corroboration. Until such validation is obtained, any extrapolation to clinical risk or public health implications should be approached with caution.

In summary, the evidence compiled here, from structural studies, molecular dynamics simulations, biophysical experiments, and omics datasets, indicates that the lipid components of LNPs possess intrinsic bioactivity that can contribute to longer-term cellular alterations, including effects observed with empty LNPs.

While comprehensive, this hypothesis-driven review remains primarily conceptual. Several limitations must be acknowledged:

1. Evidence hierarchy and model dependence: Most data integrated here derive from *in vitro* and murine studies employing high LNP doses and simplified cellular systems. Their pharmacodynamic and metabolic contexts differ from human administration, particularly with respect to intramuscular injection, depot kinetics, and systemic clearance. Translation to human physiology, therefore, requires caution.
2. Absence of long-term human datasets: There are currently no controlled longitudinal omics or lipidomic studies comparing vaccinated versus unvaccinated cohorts, nor studies isolating the effects of *empty* LNPs in humans. As a result, the persistence and mechanistic implications of the proposed L-DMD phenomena remain inferential.
3. Confounding from infection, comorbidities, and drug exposure: The majority of available transcriptomic and proteomic datasets were collected under non-standardized clinical conditions or concurrent illness, complicating attribution of observed molecular patterns specifically to LNP exposure.
4. Methodological gaps in membrane biology and lipid analytics: Quantitative measurement of membrane phosphoinositides, ionizable-lipid partitioning, or phospholipid-domain dynamics *in vivo* is technically challenging. Present biomarker assays (*e*.*g*., lipidomics or phospho-proteomics) lack temporal resolution and standardization across laboratories.
5. Unresolved dose, composition, and batch variability: Manufacturing differences in ionizable lipid identity, particle size distribution, and PEG density can alter biological activity. Without harmonized formulations, cross-study comparison and extrapolation remain uncertain.
6. Speculative extensions to clinical outcomes: Although pathways such as NF-*κ*B, MAPK, JAK/STAT, and mTORC1/2 are well established in stress and immune regulation, linking their modulation by LNPs to specific human disease states or chronic conditions remains speculative. No causal relationships have been established. Similarly, any sex-specific vaccine effects require validation.
7. Omics data require confirmation: Transcriptomics cannot provide information about protein activity, nor can proteomics identify the upstream signals that led to altered protein expression. Furthermore, protein functionality, including correct folding and binding regulation, must also be experimentally determined. Lipidomics should therefore be applied as a complementary tool to trace upstream signaling inputs and the resulting suspected perturbations of the plasma membrane and vesicular transport pathways (see experimental design proposal in the Discussion).
8. modRNA is based on the concept of suppressing intracellular pattern recognition: receptors: A crucial aspect of modRNA's success is that it is chemically modified (*e.g.*, m1*ψ*) to suppress pattern recognition receptors such as TLRs, thereby preventing premature mRNA degradation and increasing translation efficiency through nucleoside modifications^333^, ^334^, ^335^, ^336^. At the same time, however, TLRs are functionally cross-talking with LDL receptors involved in LNP uptake, as well as with the complement system^337^, ^338^, ^339^, ^340^. In addition, TLRs interact closely with membrane lipids^341^^,^^342^. This represents a potential limitation. TLR suppression (*via* modRNA modifications) combined with simultaneous TLR4 activation (*via* ionizable lipids) could produce contradictory immunological effects with additive, antagonistic, and/or synergistic consequences. This complicates attribution of observed cellular responses to the LNP structure, the modRNA payload, or their interaction.
9. Future confirmation of the L-DMD framework will require coordinated systems toxicology studies: combining standardized LNP formulations, dose-dependent multi-omic profiling, and mechanistic perturbation experiments to distinguish membrane-driven signaling effects from payload-mediated responses.

## 7 Conclusions

Collectively, structural, biophysical, and omics data indicate that LNPs possess intrinsic bioactivity that can modulate intracellular signaling independent of mRNA payload. The L-DMD framework hypothesis provides a conceptual bridge between colloidal physics and the regulation of cell systems, explaining persistence phenomena following SARS-CoV-2 mRNA vaccination, and offering practical routes for design improvement.

Lipid nanoparticles exemplify a notable intersection of colloidal chemistry and cell biology. Their interaction with biological membranes is not merely passive diffusion but rather a transient, tightly coordinated *in vivo* process governed by electrostatic charge, membrane curvature, and lipid composition. This process is further shaped by the Vroman effect and the pH-dependent protonation dynamics of ionizable lipids, leading to stochastic protein corona formation and the potential modulation of canonical and/or non-canonical signaling pathways at the membrane level. These membrane–proximal events can initiate cellular stress and metabolic signaling networks, including NF-*κ*B, MAPK, JAK/STAT, and mTOR, thereby constituting one of the earliest biochemical interfaces between a synthetic delivery vector and the living cell.

The concept of lipid-nanoparticle-driven membrane dynamics (L-DMD) describes how electrostatic and compositional disturbances may contribute to system-level transcriptional reprogramming. Future quantitative studies should aim to determine the resolution kinetics of these responses, helping to clarify where beneficial adaptive signaling concludes and where sustained or unintended persistence may start.

By viewing lipid nanoparticles as active participants in cellular decision-making rather than passive delivery vehicles, researchers may gain greater control over efficacy, safety, and temporal behavior, paving the way for a new generation of rationally engineered nanomedicines.

## Author contributions

Falko Seger: Conceptualization, Writing–original draft, Writing–review and editing, Visualization.

L. Maria Gutschi: Conceptualization, Writing–review and editing. Stephanie Seneff: Conceptualization, Writing–review and editing, Funding acquisition, Project administration.

## Declaration of generative AI and AI-assisted technologies in the manuscript preparation process

The authors used AI-assisted tools (ChatGPT, Claude, and AlterAI) for language refinement and rewording of author-drafted text, and Grammarly for grammar and spelling checking. No AI tool was used to generate original scientific content, data, interpretations, synthesis or conclusions. All authors reviewed and approved the final manuscript and take full responsibility for its content.

## Appendix A Supporting information

The following is the Supporting Information to this article.
