# Supplementary material for: Lipid nanoparticles as active biointerfaces: From membrane interaction to systemic dysregulation
Source: Acta Pharm Sin B. 2026 Jul 6;16(9):5674–708. doi: 10.1016/j.apsb.2026.07.001 (PMC13589937; doi:10.1016/j.apsb.2026.07.001)
Supplement: Supplementary file 1 [file mmc1.pdf]

Supporting Information for

Review

Lipid nanoparticles as active biointerfaces: From membrane interaction to systemic dysregulation

Falko Seger[a,†], L. Maria Gutschi[b,†], Stephanie Seneff[c,†,*]

[a]*Independent Researcher, Berlin 10117, Germany*

[b]*Pharmacy Consultant, Retired, Ottawa K1P 1C1, Canada*

[c]*Computer Science and Artificial Intelligence Laboratory, MIT, Cambridge, MA 02139, USA*

Received 19 December 2025; received in revised form 21 February 2026; accepted 25 March 2026

[*]Corresponding author. seneff@csail.mit.edu (Stephanie Seneff).

[†]These authors made equal contributions to this work.

Running title: Lipid nanoparticles as active biointerfaces

Table S1 Kinases/phosphatases and derivatives/substrates.

Substrate	Enzyme	Product
(a) Kinases		
PI	PI4Ks	PI4P
PI	VPS34	PI3P
PI5P	PIP4Ks	$PI(4,5)P_2$
PI3P	PIKfyve	$PI(3,5)P_2$
PI4P	PIPKIs	$PI(4,5)P_2$
PI3P/PI4P	Class II PI3Ks	$PI(3,4)P_2$
(b) Phosphatases		
PI4P	SAC1	PI
PI4P	SAC2	PI
PI3P	MTMs	PI
$PI(3,5)P_2$	MTMs	PI5P
$PI(3,5)P_2$	FIG4	PI3P
$PI(4,5)P_2$	SKIP	PI4P
$PI(4,5)P_2$	INPP5E	PI4P
$PI(4,5)P_2$	Synaptojanin 1/2	PI4P
$PI(4,5)P_2$	OCRL	PI4P
$PI(3,4)P_2$	INPP4A/B	PI3P
$PI(3,4)P_2$	TPIP	PI4P
$PI(3,4)P_2$	PIPP	PI4P
$PI(3,4)P_2$	SHIP1/2	PI4P
$PI(3,4)P_2$	OCRL	PI4P
$PI(3,4,5)P_3$	TPIP	$PI(4,5)P_2$
$PI(3,4,5)P_3$	PIPP	$PI(4,5)P_2$
$PI(3,4,5)P_3$	SHIP1/2	$PI(3,4)P_2$
$PI(3,4,5)P_3$	OCRL	$PI(3,4)P_2$
$PI(3,4)P_2$	PTEN	PI4P
$PI(3,4,5)P_3$	PTEN	$PI(4,5)P_2$

(a) Kinases catalyze the phosphorylation of phosphoinositides, generating specific products that regulate membrane trafficking, signaling, and cellular localization. (b) Phosphatases reverse these reactions by dephosphorylating phosphoinositides, thus maintaining phosphoinositide homeostasis. Data summarized from Posor et al.[1]

1. Mechanistical example for kinase interaction: PIP4K-mediated synthesis of $PI(4,5)P_2$ from PI5P

Phosphatidylinositol-5-phosphate (PI5P) is phosphorylated at the D-4 position of the inositol ring by phosphatidylinositol-5-phosphate-4-kinases (PIP4Ks). Following electrostatic recruitment of the enzyme to the negatively charged membrane surface, an Mg^{2+}-coordinated ATP binds to the active site. A catalytic base deprotonates the 4-hydroxyl group of PI5P, generating a nucleophilic alkoxide that performs an in-line phosphoryl transfer to the γ-phosphate of ATP. The pentacoordinated transition state is stabilized by Mg^{2+} and basic amino acid residues, resulting in the release of ADP and the formation of phosphatidylinositol-4,5-bisphosphate [$PI(4,5)P_2$]. The additional phosphorylation increases the membrane's negative surface charge and promotes the recruitment of polybasic effector proteins and downstream signal transduction processes[2].

2. Example of phosphatases: TPIP-mediated dephosphorylation of PI(3,4)P2 to PI4P

Phosphatidylinositol-3,4-bisphosphate [$PI(3,4)P_2$] is dephosphorylated by the phosphatase TPIP (specifically, the TPIP isoform) at the D-3 position of the inositol ring. After membrane recruitment *via* the N-terminal transmembrane domains, the substrate binds to the enzyme's catalytic pocket. In the catalytic motif characteristic of the PTEN family, the cysteine residue (nucleophile) acts as the primary agent: the sulfur atom of the cysteine performs a nucleophilic attack on the phosphorus atom of the 3-phosphate group, forming a phospho-cysteine intermediate. A conserved aspartate residue acts as a general acid and protonates the remaining oxygen on the inositol ring to enable the release of PI_4P. The covalent intermediate is subsequently hydrolyzed by an activated water molecule, releasing inorganic phosphate and regenerating the enzyme. The removal of the 3-phosphate group reduces the negative charge of the membrane and terminates the recruitment of 3-phosphate-specific effectors (such as TAPP1/2), which controls signal transduction.[3]

Abbreviations: Class II PI3Ks, class II phosphoinositide 3-kinases; FIG4, FIG4 phosphoinositide 5-phosphatase (SAC3);INPP4A/B, inositol polyphosphate-4-phosphatases A/B; INPP5E, inositol polyphosphate-5-phosphatase E; MTMs, myotubularin family phosphatases; OCRL, oculocerebrorenal syndrome of Lowe protein inositol polyphosphate 5-phosphatase; PI, phosphatidylinositol; $PI(3,4)P_2$, phosphatidylinositol 3,4-bisphosphate; $PI(3,4,5)P_3$, phosphatidylinositol 3,4,5-trisphosphate; $PI(3,5)P_2$, phosphatidylinositol 3,5-bisphosphate;

$PI(4,5)P_2$, phosphatidylinositol 4,5-bisphosphate; PI3P, phosphatidylinositol 3-phosphate; PI4Ks, phosphatidylinositol 4-kinases; PI4P, phosphatidylinositol 4-phosphate; PI5P, phosphatidylinositol 5-phosphate; PIKfyve, FYVE finger-containing phosphoinositide kinase; PIP4Ks, phosphatidylinositol 5-phosphate 4-kinases; PIPKIs, Type I phosphatidylinositol phosphate kinases (PI4P 5-kinases); PIPP, proline-rich inositol polyphosphate 5-phosphatase; PTEN, phosphatase and tensin homolog; SAC1, SAC domain-containing phosphatase 1; SAC2, SAC domain-containing phosphatase 2; SHIP1/2, SH2-containing inositol phosphatases 1/2; SKIP, skeletal muscle and kidney-enriched inositol phosphatase; Synaptojanin 1/2, synaptojanin 1/2 (inositol polyphosphate 5-phosphatases); TPIP, TPTE and PTEN homologs inositol lipid phosphatase; VPS34, vacuolar protein sorting 34 (class III PI 3-kinase).

References

1. Posor Y, Jang W, Haucke V. Phosphoinositides as membrane organizers. *Nat Rev Mol Cell Biol* 2022; **12**:797–816.
2. Clarke JH, Irvine RF. Evolutionarily conserved structural changes in phosphatidylinositol 5-phosphate 4-kinase (PI5P4K) isoforms are responsible for differences in enzyme activity and localization. *Biochem J* 2013; **454**: 49–57.
3. Walker SM, Downes CP, Leslie NR. TPIP: a novel phosphoinositide 3-phosphatase. *Biochem J* 2001; **360**: 277–83.
